# Lipid Metabolism–Related Genes Define Prognosis and Therapeutic Targets in Thyroid Cancer

**DOI:** 10.1155/ijog/9661210

**Published:** 2026-05-17

**Authors:** Tianze Wang, Boyuan Nan, Qila Sa, Ning Xu, Wei Zhang

**Affiliations:** ^1^ Department of Hepatobiliary, Pancreatic, Spleen, and Thyroid Surgery, General Hospital of Northern Theater Command of the Chinese People’s Liberation Army, Shenyang, 110000, China; ^2^ Department of General Surgery, Taizhou People’s Hospital Affiliated to Nanjing Medical University, Taizhou, China

**Keywords:** lipid metabolism, prognostic markers, single-cell analysis, thyroid cancer

## Abstract

**Background:**

Altered lipid metabolism is increasingly recognized as a driver of tumor progression, yet its specific role in thyroid cancer remains unclear.

**Methods:**

We combined Mendelian randomization with transcriptomic and single‐cell analyses to identify lipid‐related factors that contribute to thyroid cancer development. A lipid‐metabolism gene signature was constructed to evaluate its prognostic value, and functional assays were used to examine the biological role of ACBD7.

**Results:**

Lipid‐related metabolites showed genetic evidence of causal relevance to thyroid cancer. We identified a set of lipid‐metabolism genes that were consistently dysregulated in tumors and developed a nine‐gene signature that stratified patient prognosis with high accuracy. The high‐risk group displayed stronger lipid pathway activation, a distinct immune landscape, and a higher *BRAF* mutation rate. ACBD7 knockdown reduced thyroid cancer cell growth, invasion, and migration, suggesting a functional link between lipid metabolism and tumor aggressiveness.

**Conclusions:**

Lipid metabolic dysregulation contributes to thyroid cancer progression and may help refine clinical risk assessment. The nine‐gene signature and the functional relevance of ACBD7 highlight the potential of lipid‐targeted strategies in future therapeutic development.

## 1. Introduction

Thyroid cancer is the most common malignancy of the endocrine system, with increasing incidence rates globally. Papillary thyroid carcinoma (PTC) represents the majority of thyroid cancer cases, with anaplastic thyroid carcinoma (ATC) being one of the most aggressive and fatal forms [1]. Epidemiological studies reveal that while the prognosis for differentiated thyroid cancers such as PTC is relatively favorable, treatment options for advanced or refractory cases, including ATC, are limited [2]. Despite advancements in surgery, radioiodine therapy, and chemotherapy, these treatment modalities often fail in metastatic or aggressive thyroid cancers [3]. There remains a substantial need for identifying novel molecular markers and potential therapeutic targets.

Single‐cell sequencing technology has emerged as a powerful tool for identifying molecular markers associated with various cancer types, including thyroid cancer [4]. This technology allows for high‐resolution analysis of individual cell populations within tumors, providing insights into tumor heterogeneity and the identification of novel therapeutic targets. As explored in studies such as “Single‐cell transcriptomic analysis of the tumor ecosystems underlying initiation and progression of papillary thyroid carcinoma” [5] and “CREB3L1 promotes tumor growth and metastasis of anaplastic thyroid carcinoma by remodeling the tumor microenvironment [6],” the complex interplay between tumor cells and their microenvironment significantly influences disease progression. The combination of single‐cell sequencing data with large genomic datasets such as The Cancer Genome Atlas (TCGA) has facilitated the discovery of key genes involved in lipid metabolism. By leveraging data from single‐cell sequencing and the Gene Expression Omnibus (GEO) database, researchers have been able to identify genes that regulate metabolic pathways linked to cancer progression. Studies such as “Lipid metabolism‐related gene expression in the immune microenvironment predicts prognostic outcomes in renal cell carcinoma [7]” and “Development and validation of a novel lipid metabolism‐related gene prognostic signature and candidate drugs for patients with bladder cancer [8]” provide strong evidence that lipid metabolism plays a crucial role in the tumor microenvironment and can predict patient outcomes. These findings suggest that lipid metabolism–related genes may serve as effective biomarkers and potential therapeutic targets in thyroid cancer as well.

Although lipid metabolic reprogramming has been described in several solid tumors, its causal relevance and cell‐type specificity in thyroid cancer remain unclear. Prior studies mainly relied on bulk transcriptomic associations and could not separate correlation from causation or link metabolic changes to specific tumor or stromal compartments.

Although ARSI was predominantly annotated within CAF clusters in the single‐cell dataset, its bulk expression pattern in Figure 1 appears epithelial‐enriched. This difference likely reflects the distinct resolution of the two data types. In the scRNA‐seq dataset, ARSI expression is concentrated in a subset of matrix‐producing fibroblasts, whereas in bulk RNA‐seq, tumor epithelial cells dominate the overall transcriptomic signal, leading to an apparent epithelial pattern. In addition, CAF‐derived ARSI may colocalize with epithelial regions in bulk tissue sections due to close spatial proximity within the tumor architecture. Thus, the two findings are not contradictory; rather, they indicate that ARSI expression arises mainly from CAFs at the single‐cell level but becomes epithelial‐weighted in bulk profiles because of sample composition.

FIGURE 1Construction and evaluation of the lipid‐related prognostic model. (a) LASSO coefficient profiles of candidate genes plotted against log (λ), illustrating the shrinkage trajectories as the penalty increases. (b) Ten‐fold cross‐validation curve used to determine the optimal λ value, with error bars representing the partial likelihood deviance. (c) Kaplan–Meier survival curves for high‐ and low‐risk groups in the TCGA–THCA training cohort based on the 9‐gene signature. TCGA–THCA cohort samples were divided into training and validation sets in a 7:3 ratio. (d) RTime‐dependent ROC curves assessing the predictive performance of the 9‐gene model in the training cohort, with AUC values of 0.84, 0.84, and 0.87 at 1, 3, and 5 years, respectively. (e) Kaplan–Meier curves validating the prognostic stratification in the external validation cohort. (f) Time‐dependent ROC curve for the validation cohort, showing an AUC of 0.94 at 5 years.

**Figure:**
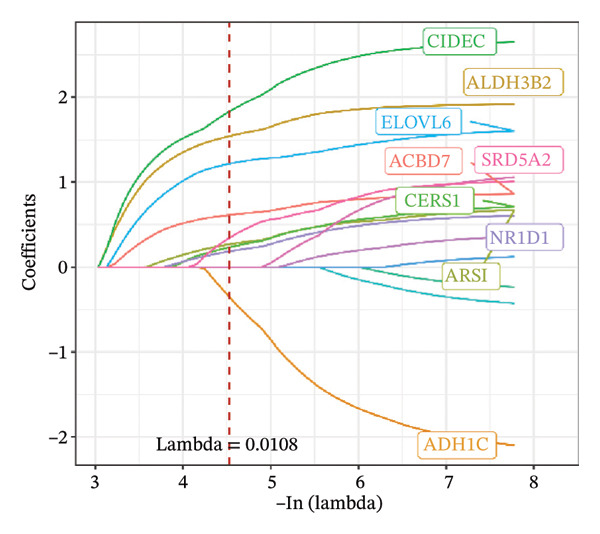
(a)

**Figure:**
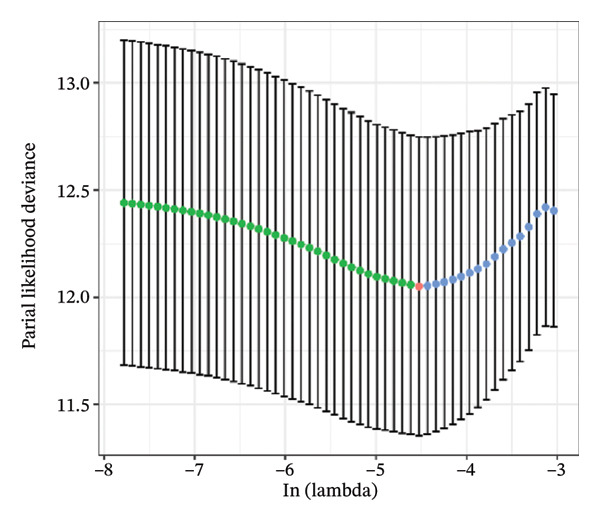
(b)

**Figure:**
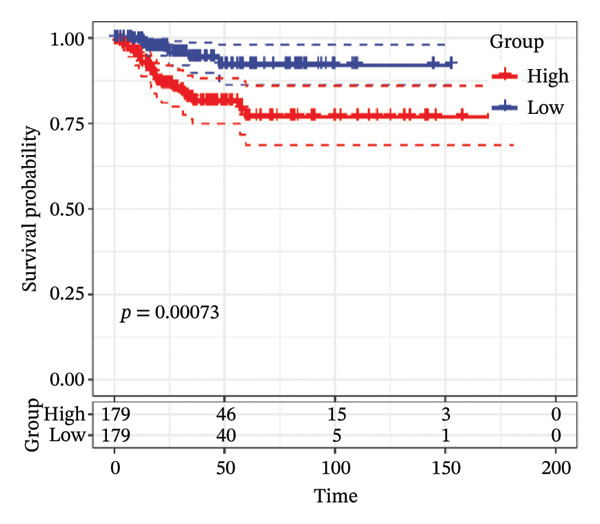
(c)

**Figure:**
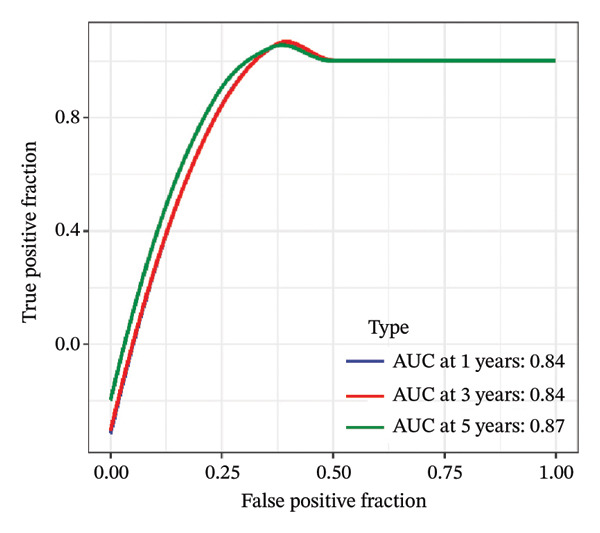
(d)

**Figure:**
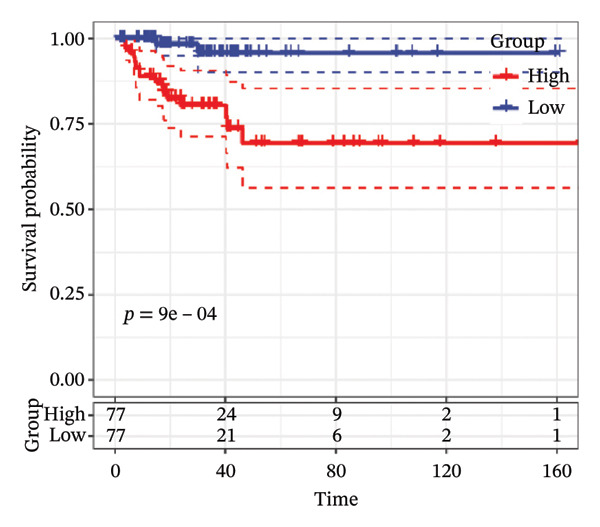
(e)

**Figure:**
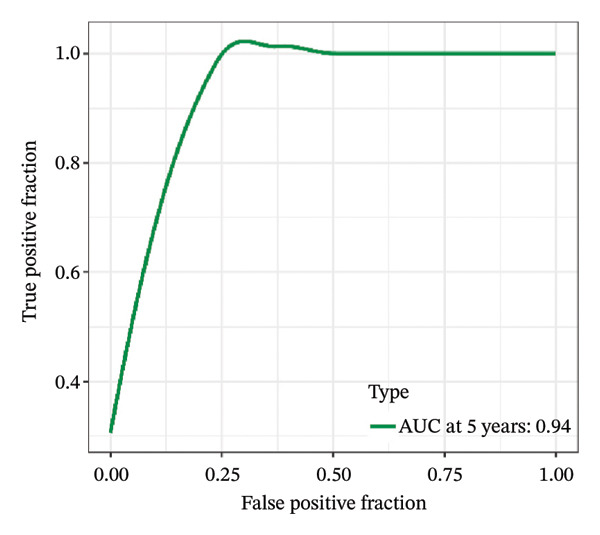
(f)

To address this gap, our study integrates MR with transcriptomic and single‐cell data to identify lipid‐related factors that contribute to thyroid cancer development. This approach allows us to pinpoint causal lipid metabolites, define a metabolism‐based gene signature with prognostic value, and reveal cell populations in which these genes are active. By combining population genetics with molecular profiling, our study provides new evidence for the role of lipid metabolism in thyroid cancer and offers biomarkers with translational potential.

Several important questions around lipid metabolism in thyroid cancer remain unanswered. Most studies have focused on expression differences and pathway enrichment, but they have not determined whether lipid alterations are causal or which cell populations drive these metabolic changes. Conventional bulk transcriptomic approaches mix signals from tumor cells and stromal components, making it difficult to define cell‐type–specific metabolic programs.

By combining MR with single‐cell sequencing, our study overcomes these limitations. MR provides genetic evidence to infer causality and reduces bias from tumor heterogeneity, while scRNA‐seq reveals where lipid‐related genes are active within the tumor ecosystem. This integrative design allows us to link causal metabolites to specific cellular contexts.

In addition, the nine‐gene signature generated from this framework has distinct prognostic value. It captures metabolic features that are not reflected by common genomic markers and can stratify patients even within clinically similar subgroups. This highlights its potential for refining risk assessment in thyroid cancer.

## 2. Methods

### 2.1. Source of GWAS Data

GWAS data for human blood metabolites were obtained from the most comprehensive genome‐wide association analysis of blood metabolites to date by Shin et al. This study included 7824 adults from Europe, 1768 from Germany and 6056 from the United Kingdom [9]. Approximately 20,000 SNPs in the population were analyzed for correlation by untargeted metabolomics GWAS. Ultimately, 447 metabolites were successfully obtained, which were grouped into nine classes (carbohydrates, lipids, amino acids, nucleotides, energy products, cofactors and vitamins, peptides, and exogenous elements). Genetic information for human blood metabolites was obtained from the Metabolomics GWAS server (https://metabolomics.helmholtz-muenchen.de/gwas/). GWAS summary statistics for thyroid tumors were obtained from the GWAS catalog (https://www.ebi.ac.uk/gwas/home) for thyroid tumor GWAS study data number GCST90399736 [10]. This study was conducted in a European population involving a total of 1339769 individuals, including 6015 patients with bladder tumors and 1333754 control participants.

### 2.2. Selection of Instrumental Variables

A series of steps were then performed to filter out instrumental variables related to blood metabolites. First, a threshold of *p* < 10 − 5 was set to extract instrumental variables from 447 metabolites. In addition, a linkage disequilibrium threshold of *r*2 < was set at 0.001, and the aggregation window size was 10,000 kb. To prevent bias using weak instrumental variables, the F statistic for each metabolite was calculated to analyze the efficacy of the instrumental variables. To ensure that all SNPs had sufficient variance for the metabolite of interest, weak instrumental variables (instrumental variables with *F* < 10) were eliminated. Exposure‐related SNPs were then extracted from the outcome data, and SNPs with a significant increase (*p* < 5 × 10^−8^) in association with the outcome were removed. Subsequent coordination was performed to ensure that SNP exposure alleles and SNP results were consistent, and palindromic SNPs were discarded.

### 2.3. Mendelian Randomization (MR) and Sensitivity Analyses

The main MR method used to determine the causal relationship between exposure and outcome is the inverse variance weighted (IVW) model. IVW is an efficient method of analysis based on the assumption that all genetic variants are valid instrumental variables. IVW provides robust and consistent estimates of the causal effect of exposure on outcome when genetic variation satisfies the three instrumental variable assumptions and is not subject to multiplicity. MR–Egger, MR–Egger (bootstrap), Simple mode (NOME), Weighted median, and Weighted mode complement the MR approach. Complementary to the MR approach, these methods use different hypothetical models to assess causality. The directional consistency of the different MR analytical models enhances the credibility of causal inferences. In addition, Cochran’s *Q*‐test analysis was used to assess heterogeneity, which was considered to be present if the *Q*‐test had a *p* < 0.05. In addition, the intercept in the MR–Egger regression is a valid indicator of response to horizontal polytropy, which can be calculated by mr_pleiotropy_test, If *p* < 0.05, horizontal polytropy is considered to affect IVW, indicating that the findings are not reliable. To reduce confounding arising from tumor stage heterogeneity, the thyroid cancer GWAS used in the MR analysis was restricted to differentiated thyroid carcinoma. Undifferentiated and anaplastic tumors were not included in the outcome GWAS, minimizing bias from mixed‐stage or mixed‐lineage disease. Because MR relies on germline variants determined before disease onset, the genetic instruments are inherently independent of clinical stage at diagnosis, providing additional protection against confounding from advanced or poorly differentiated cases. Subsequently, leave‐one‐out analyses were performed to assess whether the significant results were caused by specific instrumental variables, and the MR Steiger directionality test was performed to explore the correctness of the causal direction. Finally, funnel plots and scatter plots were available to complement the sensitivity assessment. All data analyses for MR were done using the R package TwoSampleMR (Version 0.5.8) [11].

### 2.4. Identification of Lipid Metabolism–Related Differential Genes

Firstly, we collected 853 genes related to lipid metabolism from the KEGG (https://www.genome.jp/kegg/) [12] database and the Reactome (https://reactome.org/) [13] database. Subsequently, we downloaded the clinical phenotype data of thyroid cancer from the UCSC Xena (https://xena.ucsc.edu/) database. Due to the long survival period of thyroid cancer patients, we chose to use the progression‐free interval (PFI) as an indicator for assessing patient prognosis. In addition, we downloaded gene expression data of TCGA–THCA from The TCGA (https://www.cancer.gov/ccg/), which resulted in 513 tumor samples and 59 normal control samples. In total, we identified the expression of 844 lipid metabolism–related genes in the gene expression data of TCGA–THCA. We performed differential analysis of these genes using DESeq2 (Version 1.42.1) [14] software and identified significantly differentially expressed genes using |log_2_FoldChange| > 1 and padj < 0.05 as thresholds.

### 2.5. Lipid Metabolism–Related Prognostic Model Construction

First, we performed one‐way Cox regression analysis on the screened differential lipid metabolism genes in the TCGA–THCA cohort and used *p* < 0.05 as the screening threshold. Then, we divided the TCGA–THCA cohort samples into training and validation sets in a 7:3 ratio. Based on the screened differential lipid metabolism–related prognostic genes, we performed Lasso–Cox regression analyses using the glmnet package (Version 4.1–6) [15] in the training set to identify the key genes and model the prognostic risk profile. The formula for this model is RiskScore = Σ(*αi* × *βi*), where *αi* denotes the regression coefficient for each gene and *βi* denotes the gene expression value. We categorized patients into high‐ and low‐risk groups based on the median value of the RiskScore and performed Kaplan–Meier (KM) analysis using the survminer package (Version 0.4.9) [16] to describe and compare the survival curves of the two risk groups. Also, we used the timeROC package (Version 0.4) [17] in R to assess the accuracy of the risk model by receiving the area under the operating characteristic curve (ROC). To validate the accuracy of the model, we applied the model to the validation set and validated it using both KM curves and ROC curves as well.

### 2.6. Gene Expression Characteristics Between Risk Groups

In order to identify the gene expression characteristics between the high‐risk and low‐risk groups in the model, we performed a differential analysis of the gene expression data of these two groups. All genes were analyzed using DESeq2, and |log_2_FoldChange| > 1 and padj < 0.05 were set as screening thresholds to identify significantly differentially expressed genes.

### 2.7. Enrichment Analysis

To determine the pathways and functions involved in the differential genes we identified in the two differential analyses, we performed Gene Ontology (GO, https://geneontology.org) [18] using the clusterProfiler package (Version 4.2.2) [19] and Kyoto Encyclopedia of Genes and Genomes (KEGG) enrichment analyses to reveal the functions of these genes. In addition, to explore the relationship between high‐risk and low‐risk groups and lipid metabolism pathways, we performed gene set enrichment analysis (GSEA) based on 14 lipid metabolism–related pathways in the KEGG database [20].

### 2.8. Mutational Profiles Among Risk Groups

We used the TCGAbiolinks package (Version 2.24.3) [21] to download the single nucleotide variant (SNV) mutation information of the TCGA–THCA gene computed by Mutect2 software. The Homologous Recombination Defects, Fraction Altered of TCGA–THCA samples, Number of Segments, Nonsilent Mutation Rate, Aneuploidy Score, and Silent Mutation Rate data were then obtained from a previous study [22].

### 2.9. Landscape of Immune Cell Infiltration Between Risk Groups

To further understand the relationship between the model and tissue immune cell infiltration, we used the IOBR package (Version 0.99.9) [23] to calculate the immune infiltration obtained by eight immune infiltration assessment algorithms (including MCPcounter, EPIC, IPS, xCell, CIBERSORT, QUANTIseq, ESTIMATE, and TIMER) data and visualized the results as heatmaps to depict the immune landscape of different subtypes. At the same time, we calculated the correlation of the different immune infiltration scores with the model scores and the genes within the model using Spearman’s algorithm and visualized the correlation results on another heatmap for comparison.

### 2.10. Drug Sensitivity of Risk Model Genes

We downloaded data containing gene expression, drug information and cell line information, from the CellMiner database (https://discover.nci.nih.gov/cellminer/) [24]. As there were some missing values in the drug sensitivity data, we used the impute.knn function in the impute package (Version 1.76.0) [25] to evaluate and complete these missing values. Subsequently, we calculated Pearson correlation coefficients between gene expression and different drugs in the risk model and screened gene and drug pairs based on thresholds of *p* < 0.05 and |cor| > 0.55.

### 2.11. Clinical Characterization and Columnar Plots

To assess whether the model could be used as an independent prognostic factor for thyroid cancer, we performed univariate and multivariate Cox regression analyses including RiskScore, age, sex, and tumor stage in both the training and validation subsets of the TCGA–THCA cohort. In addition, we combined information on patients’ age, sex, and tumor stage with RiskScore to co‐construct a new column‐line plot model to more accurately assess patients’ prognostic status. The column‐line plots were drawn using the regplot package (Version 1.1) [26].

### 2.12. Data Sources and Quality Control of Single Cells

We used the Seurat (Version 4.4.0) [27] R package to process the single‐cell dataset from the GEO database, which is single‐cell expression profiling data of thyroid cancer (GSE184362) [5], and the sequencing platform is Homo sapiens based on GPL24676 Illumina NovaSeq 6000. The dataset includes 7 cases of cancer tissues and 6 single‐cell sequencing samples of paraneoplastic tissues. We performed data filtering by requiring that each gene be expressed in at least 3 cells, with at least 250 genes expressed per cell, and calculated the percentage of mitochondrial, ribosomal, and erythroid genes using the PercentageFeatureSet function. We ensured that the number of genes expressed per cell was between 500 and 4000, the UMI number was less than 15,000, the mitochondrial gene content was less than 15%, and erythrocyte gene expression was less than 1%.

### 2.13. Cell Downscaling and Annotation

We normalized the data of 14 samples separately by log‐normalization. Highly variable genes were found by the FindVariableFeatures function (identifying variable features based on variance stabilization transformations (“vst”)), followed by scaling by using the ScaleData function for all genes, followed by PCA downscaling to find anchors by RunPCA, selecting dim = 30, and clustering cells by FindNeighbors and FindClusters functions (setting Resolution = 0.5). To avoid the batch effect on the samples, the Harmony package (Version 0.1.0) [28] was used to batch correct the samples. We performed cell type annotation by combining the HumanPrimaryCellAtlasData dataset from the SingleR (Version 1.10.0) R package [29] with the CellTypist [30] software. CellTypist annotation was performed using the “Immune_All_Low” and “Human_Lung_Immune” pretrained reference models (2023 release), which provide broad immune and stromal coverage suitable for thyroid‐tumor microenvironment analysis. Marker genes for each subpopulation were screened by the RunDEtest function in the SCP (Version 0.5.2) [31] package by fc.threshold = 1 with a corrected *p* < 0.05. Single‐cell subpopulation differential gene enrichment analysis was done using the FeatureHeatmap function in the SCP package.

### 2.14. Risk Gene Scoring

We analyzed the genes associated with lipid metabolism obtained using one‐way Cox regression and scored each cell using the AUCell (Version 1.16.0) [32] algorithm. Next, scoring thresholds for active cells were analyzed using the AUCell_exploreThresholds function. Based on these thresholds, we categorized the cells into two groups: high and low activity of risk genes. Then, for the high‐activity cells, we performed GSEA using 14 pathways related to lipid metabolism from the KEGG database.

### 2.15. Cell Culture

TPC‐1, a human thyroid papillary carcinoma cell line, was obtained from the National Collection of Authenticated Cell Cultures (Shanghai, China). The cells, confirmed by short tandem repeat (STR) authentication, were cultured in RPMI‐1640 medium supplemented with 10% fetal bovine serum (FBS), 100 U/mL penicillin, and 0.1 mg/mL streptomycin in a humidified atmosphere at 37°C with 5% CO_2_.

### 2.16. Colony Formation Assay

Cells were plated at 1 × 10^3^ cells per well in 6‐well plates and allowed to grow for a specified period. During this time, individual cells proliferated to form clusters, with groups of 30 or more cells being counted as distinct colonies.

### 2.17. Transwell Assays

Transwell assays were utilized to assess the migratory and invasive characteristics of TPC‐1 cells. For the migration assay, cells were suspended in serum‐free medium and placed in the upper chamber of a Transwell insert (Corning, NY, USA) at a concentration of 1 × 10^5^ cells in 200 μL of medium. The lower chamber was filled with 700 μL of DMEM supplemented with 20% serum, serving as a chemoattractant. After a 24‐h incubation at 37°C, nonmigratory cells were removed from the upper side of the membrane, while the migrated cells on the underside were fixed in methanol or a methanol–acetone mixture (1:1) for 30 min and stained with 0.5% crystal violet. The cells were then imaged and counted under a microscope.

For the invasion assay, a similar procedure was followed, but the membrane was precoated with Matrigel to simulate the extracellular matrix, and the incubation period was extended to 36 h. ImageJ software was used to quantify the density of cells that had migrated or invaded.

### 2.18. Statistical Analysis

All experiments were performed in triplicate or more to ensure reliability. Data are expressed as mean ± standard deviation or mean ± standard error of the mean. Statistical comparisons between two groups were performed using the *t*‐test, while comparisons among multiple groups were analyzed using one‐way ANOVA or the unpaired two‐tailed Student’s *t*‐test, followed by Bonferroni’s post hoc test. A *p* value of less than 0.05 was deemed statistically significant, and all statistical tests were two‐sided. Analyses were conducted using GraphPad Prism 9 software (USA).

## 3. Results

### 3.1. Causal Relationship Between Blood Metabolites and Thyroid Cancer

We screened more than 400 blood metabolites and generated 6309 SNPs for these metabolites for instrumental variables. All instrumental variables had F‐statistics greater than 10. Using the inverse variance weighting (IVW) method, we obtained 14 positive results that demonstrated all blood metabolites in the six analytical methods of the MR results (Figure 2(a)). Among these significant metabolites, lipid‐related metabolites were prioritized because their causal estimates were consistent across all MR models and because lipids are known regulators of thyroid and other endocrine tumor biology. These two features—statistical robustness and biological relevance—provided a clear rationale for focusing on lipid metabolism in the subsequent analyses. Forest plots specifically highlight those metabolites with a causal relationship and specify effect sizes and confidence intervals (Figure 2(b)). Pie charts showed the main categories of these 14 metabolites, and we found that the largest proportion of metabolites associated with thyroid cancer were lipids (Supporting Figures 1–5 and Figure 2(c)).

FIGURE 2Identification of thyroid cancer–associated metabolites using Mendelian randomization analyses. (a) Heatmap displaying the *p* values of all 400 metabolites across 4 MR methods (IVW, MR–Egger, weighted median, sample mode, and weighted mode). Metabolites highlighted in red on the right are those meeting the IVW significance threshold (*p* < 0.05). (b) Forest plot summarizing causal estimates for the 14 metabolites that remained significant, with results shown across different MR algorithms for comparison. (c) Pie chart illustrating the category distribution of these 14 metabolites based on their biochemical classifications.

**Figure:**
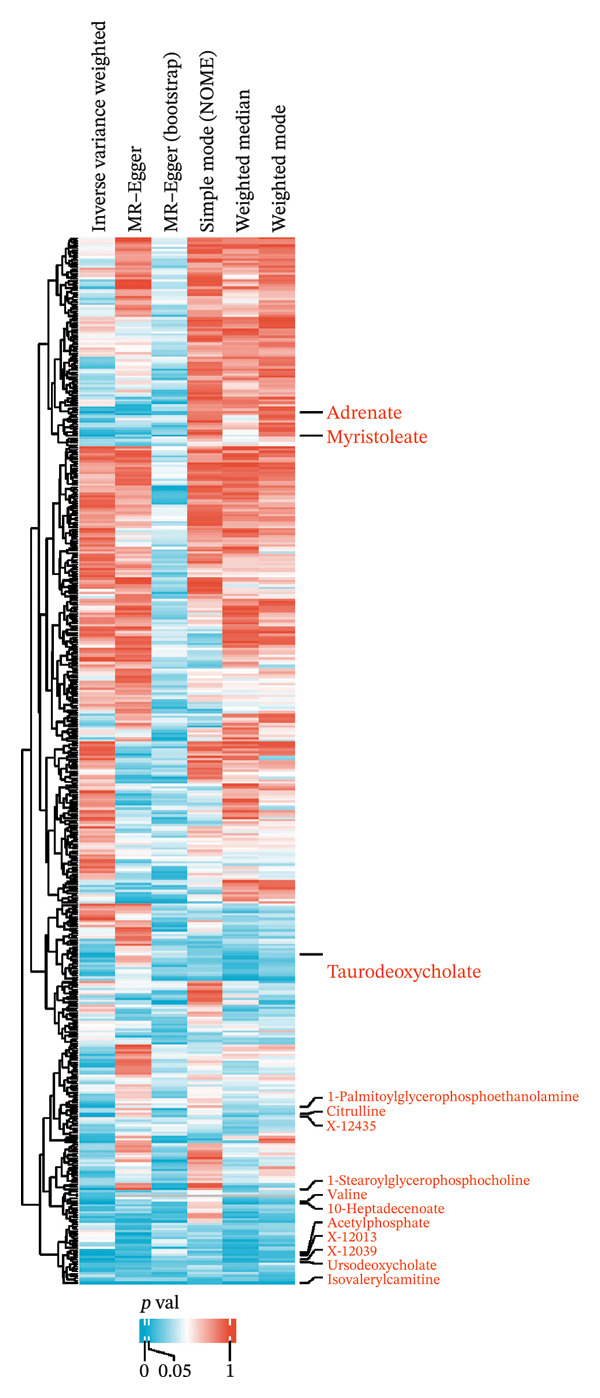
(a)

**Figure:**
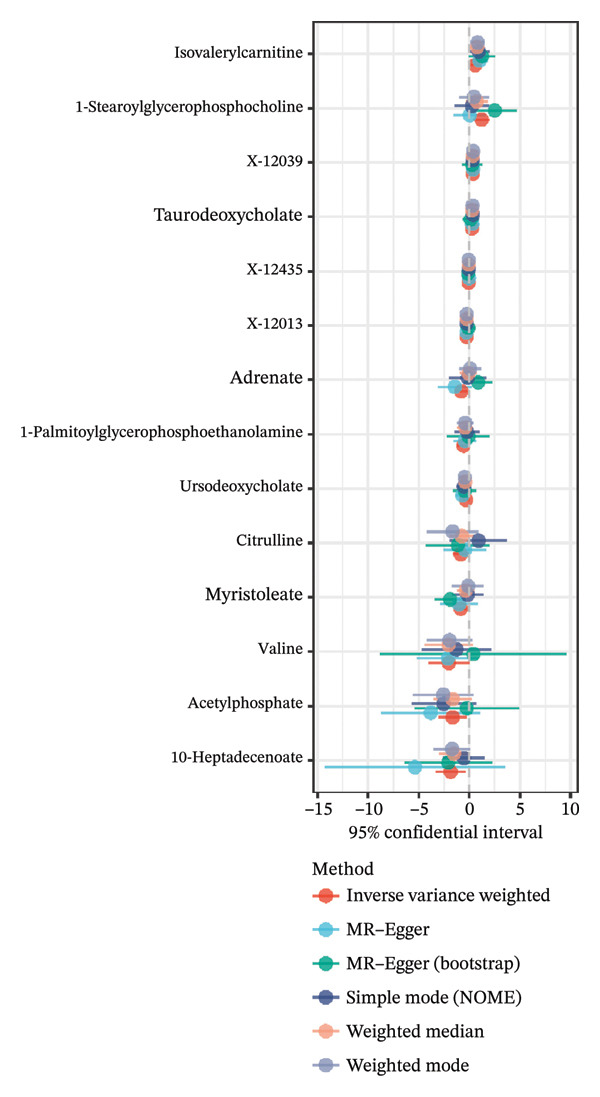
(b)

**Figure:**
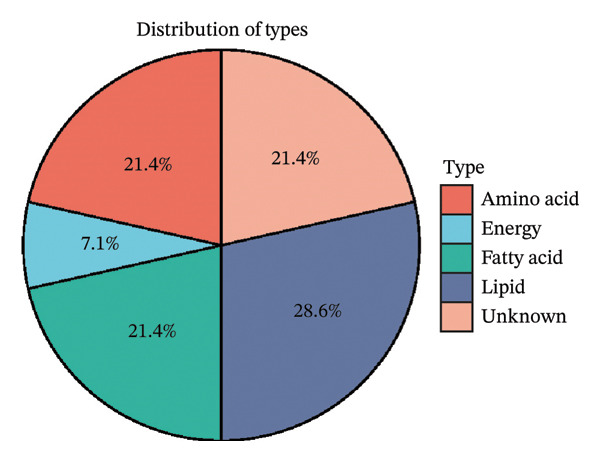
(c)

### 3.2. Identification of Lipid Metabolism–Related Differential Genes

Through differential analysis of lipid metabolism–related genes collected from the database, we identified a total of 80 upregulated lipid metabolism genes, for example, LIPH, PLA2G12B, and CHAT, and 40 lipid metabolism downregulated genes, for example, PLA2R1, UGT2B11, and ADH1B (Figures 3(a) and 3(b)). We then performed GO enrichment analysis on these genes and visualized the respective top 5 entries of BP, CC, and MF, respectively. The top 5 entries enriched for BP were lipid catabolic process, fatty acid metabolic process, glycolipid metabolic process, phospholipid metabolic process, and steroid metabolic process; the top 5 entries enriched for CC were high‐density lipoprotein particle, plasma lipoprotein particle, lipoprotein particle, protein–lipid complex, and lipid droplet; MF is enriched for lipid droplet. The top 5 entries enriched for MF were calcium‐dependent phospholipase A2 activity, phospholipase A2 activity, phospholipase activity, carbohydrate ester hydrolase activity, and lipase activity (Figure 3(c)). In addition to GO enrichment analysis, we also performed KEGG enrichment analysis and visualized the top 30 pathways, with the top three most notable being glycerophospholipid metabolism, ether lipid metabolism, and arachidonic acid metabolism (Figure 3(d)).

FIGURE 3Differential expression and functional enrichment analyses of lipid‐metabolism–related genes in thyroid cancer. (a) Volcano plot of differentially expressed lipid‐metabolism–related genes in the TCGA–THCA dataset, identified using |log_2_FC| > 1 and padj < 0.05. (b) Heatmap showing the expression patterns of these genes across tumor and adjacent normal samples. (c) Circular GO enrichment plot highlighting the major biological processes (BP), cellular components (CC), and molecular functions (MF) associated with the differential genes. (d) KEGG pathway enrichment results for the same gene set, displayed as a bar plot.

**Figure:**
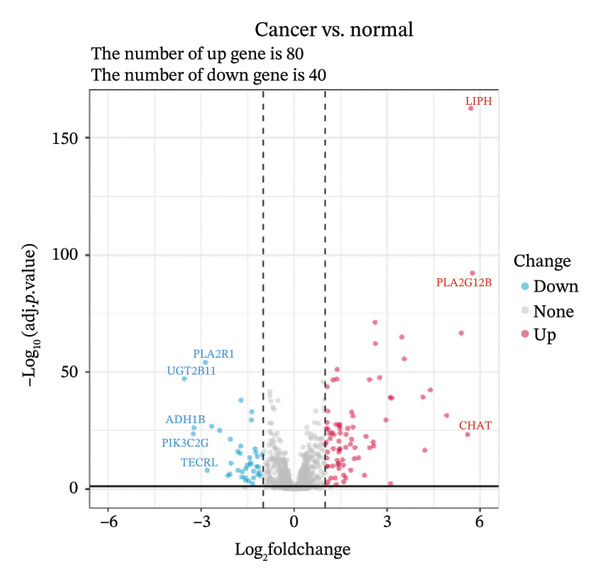
(a)

**Figure:**
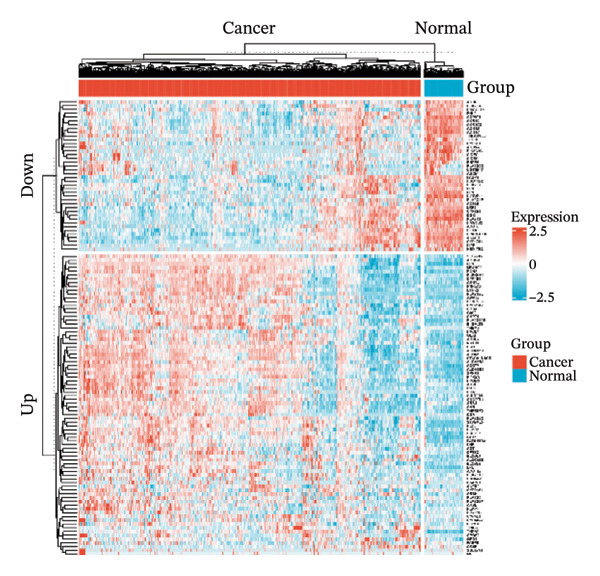
(b)

**Figure:**
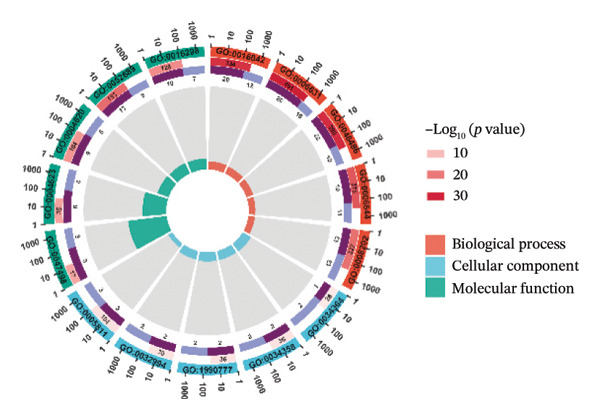
(c)

**Figure:**
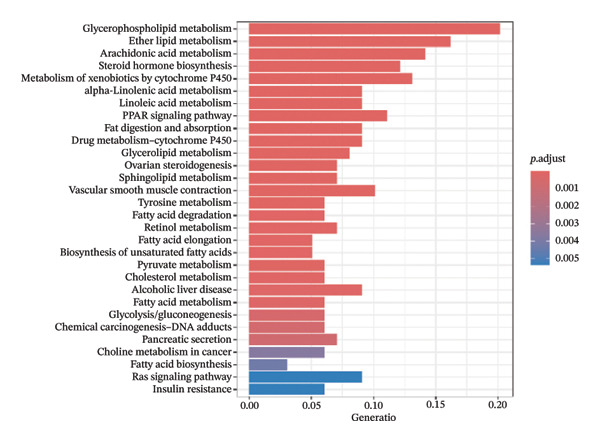
(d)

### 3.3. Prognostic Modeling

We performed a one‐way Cox analysis of differential lipid metabolism genes using PFI and screened a total of 14 genes, including 10 prognostic risk genes and 4 prognostic protection genes (see Supporting Figure 5 for details). To further optimize the risk model, we performed feature selection for these 14 genes using LASSO regression to reduce the number of genes. First, we analyzed the trajectory of each independent variable (Figure 1(a)), and it could be observed that the coefficients of the independent variables gradually converged to 0 as the lambda value increased. Ten‐fold cross‐validation was used to construct the model, and the confidence intervals under each lambda were analyzed (Figure 1(b)). The results show that the model performs best when lambda = 0.0108. Therefore, we chose the nine genes screened at lambda = 0.0108 as the target genes for subsequent analyses. These 9 genes were NR1D1, ALDH3B2, ELOVL6, ACBD7, ARSI, CIDEC, CERS1, ADH1C, and SRD5A2. Ultimately, we constructed a 9‐gene signature RiskScore formula based on these 9 genes as follows: RiskScore = 0.395 ∗ NR1D1 + 1.8085 ∗ ALDH3B2 + 1.3851 ∗ ELOVL6 + 0.744 ∗ ACBD7 + 0.422 ∗ ARSI + 2.5501 ∗ CIDEC + 0.4992 ∗ CERS1 − 1.8552 ∗ ADH1C + 0.9119 ∗ SRD5A2. We calculated the training and validation sets on the RiskScore for each sample and evaluated the classification efficiency of prognostic prediction at 1, 3, and 5 years. In the training set, the AUC for 1, 3, and 5 years exceeded 0.8. Samples with a *Z*‐score greater than zero were classified as high‐risk groups, and samples less than zero were classified as low‐risk groups by a *Z*‐score normalization of the RiskScore, and KM curves were plotted. The results showed that the survival difference between high and low RiskScore groups was extremely significant (*p* < 0.001, Figures 1(c) and 1(d)). To verify the accuracy of the model, we applied the same method in the validation set, and the results were similar to the training set. Notably, the ROC curves in the validation set showed lower results in the fifth year, possibly due to the longer survival time of thyroid cancer patients and the relatively small number of samples in the training set at 1 and 3 years (Figures 1(e) and 1(f)).

### 3.4. Gene Expression Differences Between Risk Groups

We identified differential genes between high‐ and low‐risk groups and identified 129 upregulated genes (e.g., VTCN1, SPRR1B, CHAT) and 179 downregulated genes (e.g., RGS8, SLC5A5, TFF2) (Figures 4(a) and 4(b)). We first performed GO enrichment analysis on these genes, in which the five most significant entries of BP were negative regulation of endopeptidase activity, collagen catabolic process, negative regulation of hydrolase activity, negative regulation of peptidase activity, and keratinization; the five most significant entries for CC were corrupted envelope, collagen‐containing extracellular matrix, monoatomic ion channel complex, endoplasmic reticulum lumen, and collagen trimer; and the 5 most significant entries for CC were endopeptidase inhibitor activity, peptidase inhibitor activity, endopeptidase regulator activity, peptidase regulator activity, and enzyme inhibitor activity (Figures 4(c), 4(d), and 4(e)). In addition to this, we performed GSEA enrichment analysis of 14 lipid metabolic pathways from the KEGG database and found that arachidonic acid metabolism was activated while steroid hormone biosynthesis and fatty acid degradation were inhibited in the high‐risk group (Supporting Figure 7 and Figures 4(f), 4(g), 4(h)).

FIGURE 4Transcriptomic differences and functional enrichment analyses between high‐ and low‐risk groups in thyroid cancer. (a) Volcano plot showing differentially expressed genes between high‐ and low‐risk groups in the TCGA–THCA cohort, with upregulated and downregulated genes highlighted. (b) Heatmap illustrating the expression patterns of these differential genes across the two risk groups. (c) Top five enriched biological process (BP) terms from GO analysis of the differential genes. (d) Top five enriched cellular component (CC) terms. (e) Top five enriched molecular function (MF) terms. (f–h) GSEA results highlight three representative pathways enriched between risk groups: arachidonic acid metabolism (f), steroid hormone biosynthesis (g), and fatty acid degradation (h).

**Figure:**
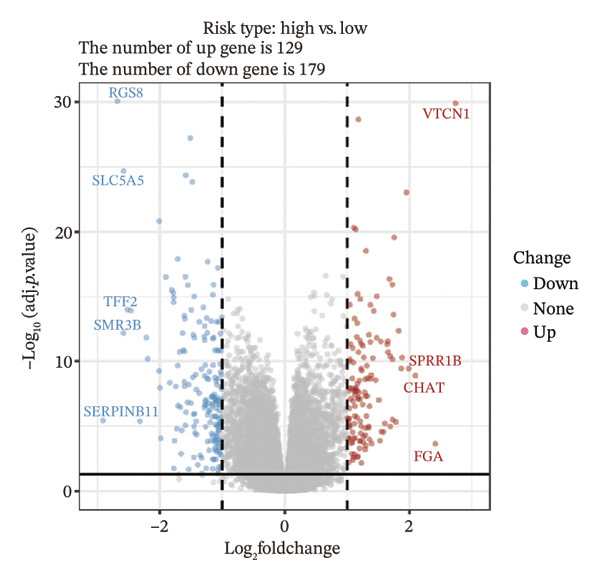
(a)

**Figure:**
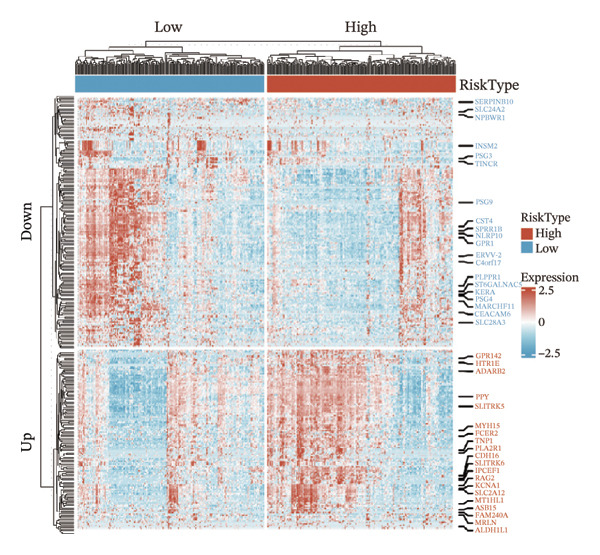
(b)

**Figure:**
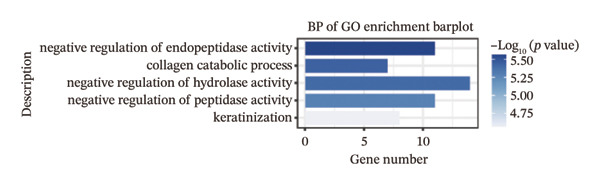
(c)

**Figure:**
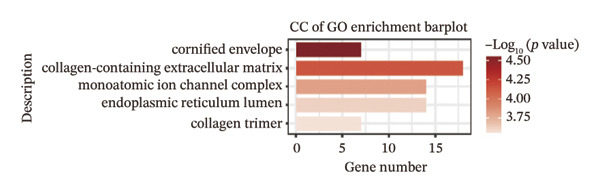
(d)

**Figure:**
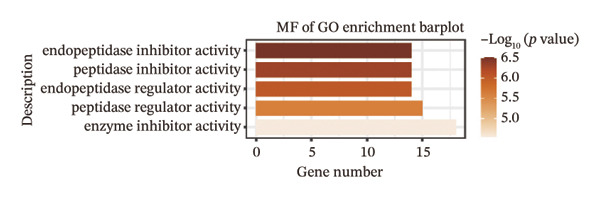
(e)

**Figure:**
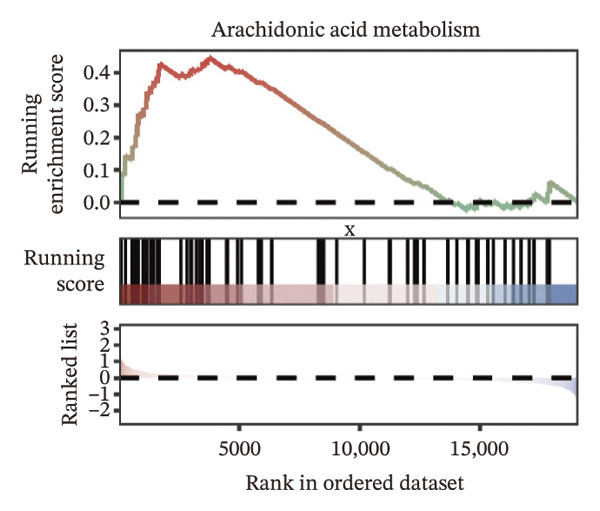
(f)

**Figure:**
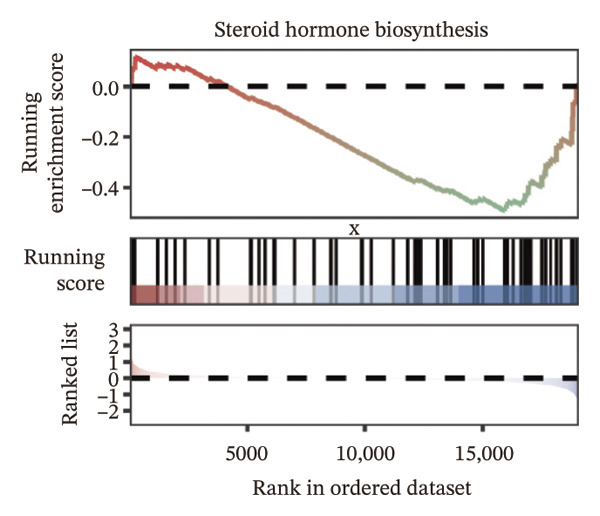
(g)

**Figure:**
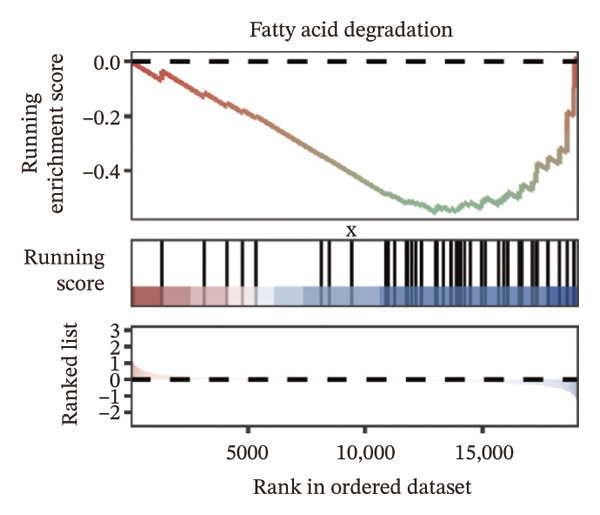
(h)

### 3.5. Mutation Characteristics of Molecular Subtypes

We obtained the SNV mutation data of TCGA by the Mutect2 tool and showed the mutations of the top 15 most significantly mutated genes in the high‐ and low‐risk groups. The analyses showed that the BRAF gene had the highest mutation frequency in thyroid cancer and that the BRAF gene was significantly more frequently mutated in the high‐risk group than in the low‐risk group (Figures 5(a) and 5(b)). In addition, we compared the distribution of the following metrics between high‐ and low‐risk groups: homologous recombination defects, fraction altered, number of segments, nonsilent mutation rate, aneuploidy score, and silent mutation rate. The results showed that the high‐risk group was higher than the low‐risk group in both nonsilent mutation rate and silent mutation rate (Supporting Figure 5 and Figure 5(c), 5(d), 5(e), 5(f), 5(g), and 5(h), reference PMC5982584).

FIGURE 5Genomic alterations between high‐ and low‐risk groups in the TCGA cohort. (a) Oncoplot showing the landscape of somatic mutations in the high‐risk group, including mutation frequency, mutation types, and base substitution patterns. (b) Oncoplot illustrating the somatic mutation profile of the low‐risk group for comparison. (c–h) Violin plots comparing genomic instability–related metrics between high‐ and low‐risk groups, including (c) homologous recombination defect (HRD) score, (d) fraction of the genome altered, (e) number of copy‐number segments, (f) nonsilent mutation rate, (g) aneuploidy score, and (h) silent mutation rate.

**Figure:**
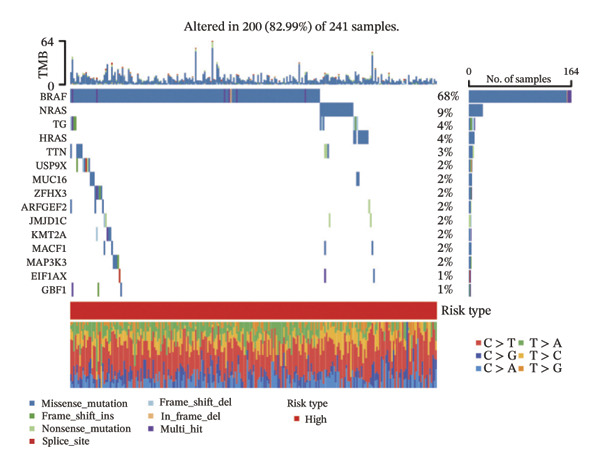
(a)

**Figure:**
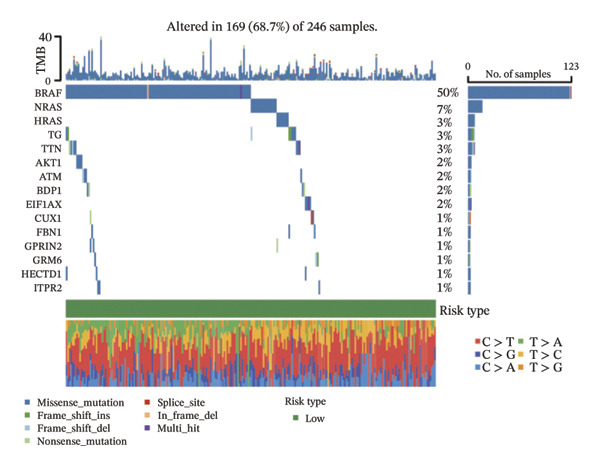
(b)

**Figure:**
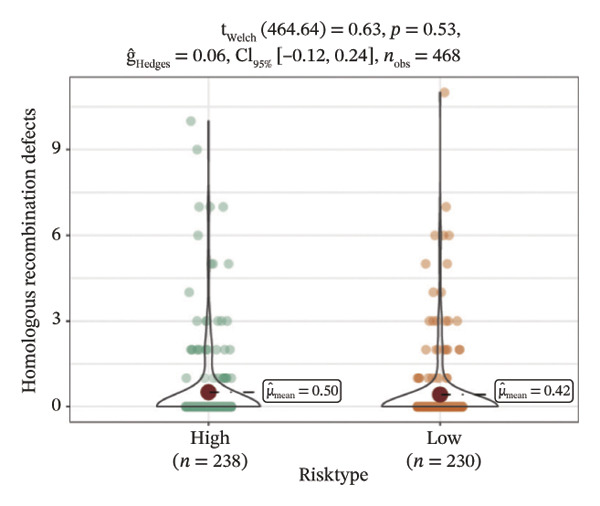
(c)

**Figure:**
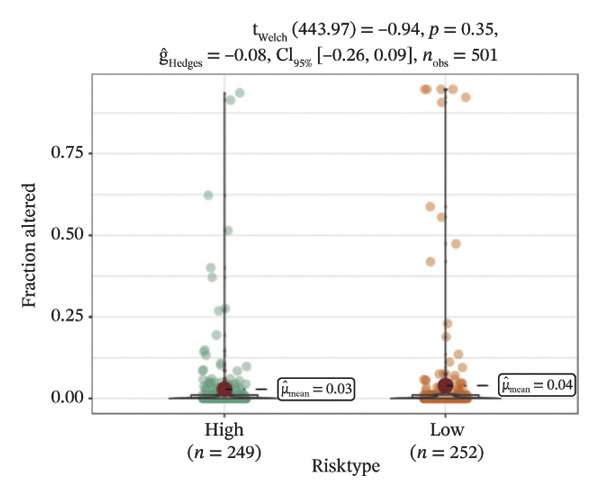
(d)

**Figure:**
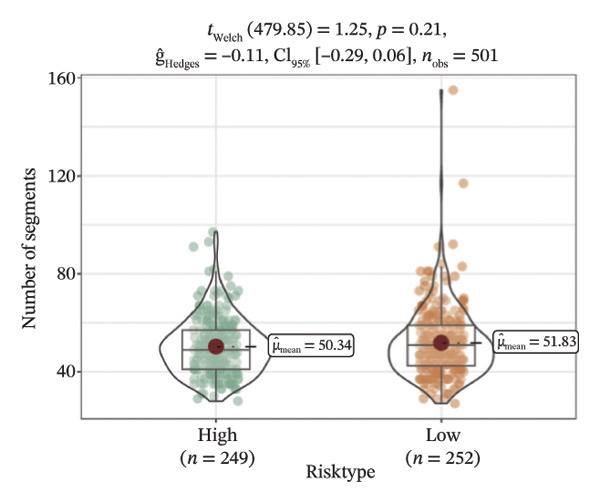
(e)

**Figure:**
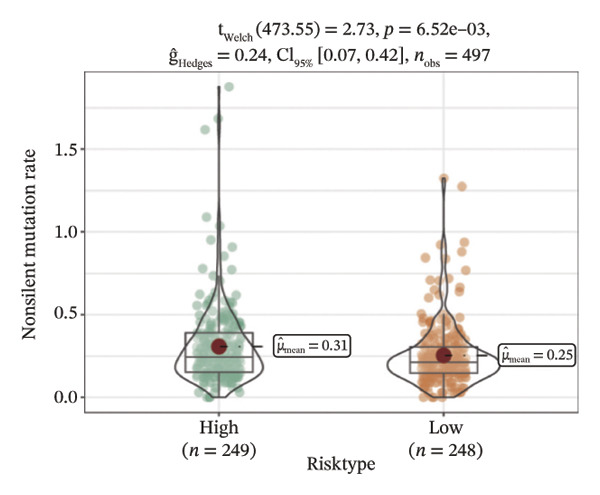
(f)

**Figure:**
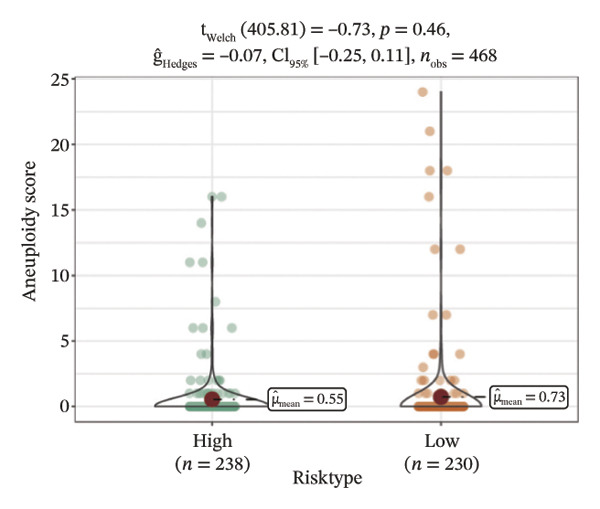
(g)

**Figure:**
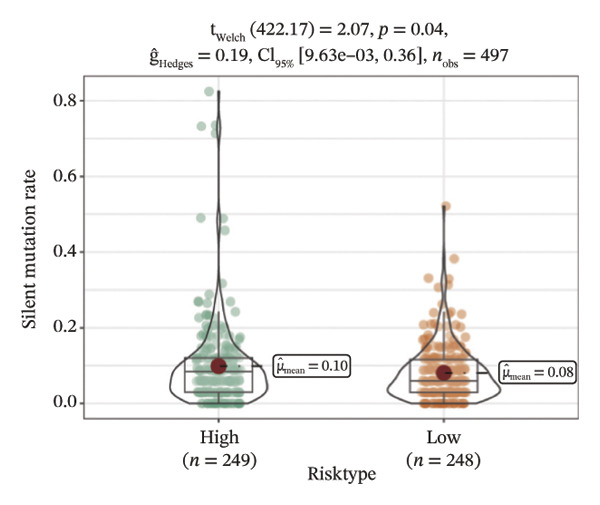
(h)

### 3.6. The Immune Landscape Between Groups

We calculated the scores of the various cell types in the tissue samples using eight different algorithms to estimate their proportions and plotted a heat map of the immune landscape according to the level of RiskScore (Figure 6(a)). Subsequently, we also calculated the correlation of RiskScore and their associated genes with the various cell types and plotted a correlation heat map (see Figure 6(j)). Interestingly, we found that ARSI among the risk model genes showed positive correlations with multiple groups of cells, with the correlations with the three most significant cell types being dendritic cells (DCs) calculated by the xCell algorithm (*R* = 0.73, *p* < 0.001, see Figure 6(b)), macrophages M1 calculated by the Quantiseq algorithm (Macrophages M1) (*R* = 0.7, *p* < 0.001, see Figure 6(c)), and cancer‐associated fibroblasts (CAFs) calculated by the EPIC algorithm (*R* = 0.69, *p* < 0.001, see Figure 6(d)). In addition, we also found that ARSI was negatively correlated with tumor purity calculated by the ESTIMATE algorithm (*R* = −0.56, *p* < 0.001, see Figure 6(e)). Unlike ARSI, SRD5A2 showed the opposite correlation. For example, SRD5A2 was negatively correlated with classical dendritic cells (cDC) (*R* = −0.61, *p* < 0.001, see Figure 6(f)) and DCs (*R* = −0.59, *p* < 0.001, see Figure 6(g)) computed by the xCell algorithm, and with CAFs (*R* = −0.58, *p* < 0.001, see Figure 6(h)) computed by the EPIC algorithm. Meanwhile, SRD5A2 was positively correlated with tumor purity calculated by the ESTIMATE algorithm (*R* = 0.49, *p* < 0.001, see Figure 6(i)).

FIGURE 6Immune infiltration characteristics associated with the risk groups and key model genes. (a) Heatmap showing immune cell infiltration patterns in high‐ and low‐risk groups across eight computational algorithms: MCPcounter, EPIC, IPS, xCell, CIBERSORT, QUANTIseq, ESTIMATE, and TIMER. (b) Positive correlation between ARSI expression and dendritic cell abundance estimated by xCell (*R* = 0.73, *p* < 0.001). (c) Positive correlation between ARSI expression and M1 macrophages based on the QUANTIseq algorithm (*R* = 0.70, *p* < 0.001). (d) Positive correlation between ARSI expression and cancer‐associated fibroblasts (CAFs) calculated by EPIC (*R* = 0.69, *p* < 0.001). (e) Negative correlation between ARSI expression and tumor purity estimated by ESTIMATE (*R* = −0.56, *p* < 0.001). (f–i) Associations between SRD5A2 expression and selected immune or stromal components, including classical dendritic cells (f), dendritic cells (g), and CAFs (h) estimated by xCell and EPIC, as well as a positive correlation with tumor purity (i). (j) Correlations between NR1D1, ALDH3B2, ELOVL6, ACBD7, ARSI, CIDEC, CERS1, ADH1C, SRD5A2 expression and tumor infiltrating immune cells.

**Figure:**
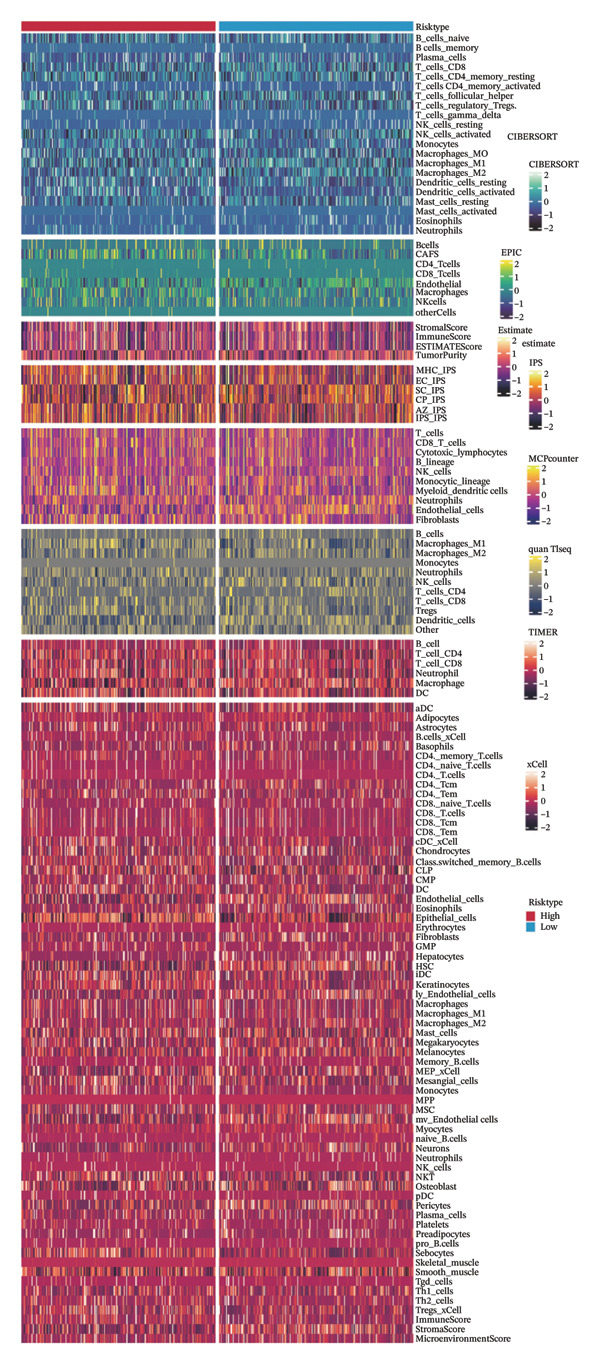
(a)

**Figure:**
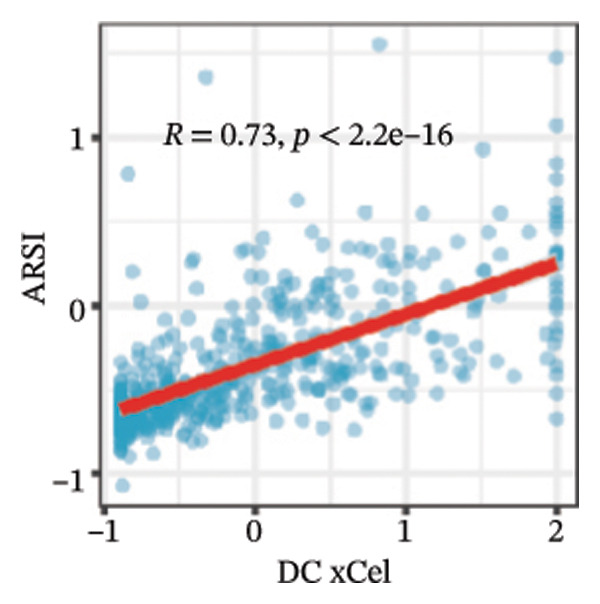
(b)

**Figure:**
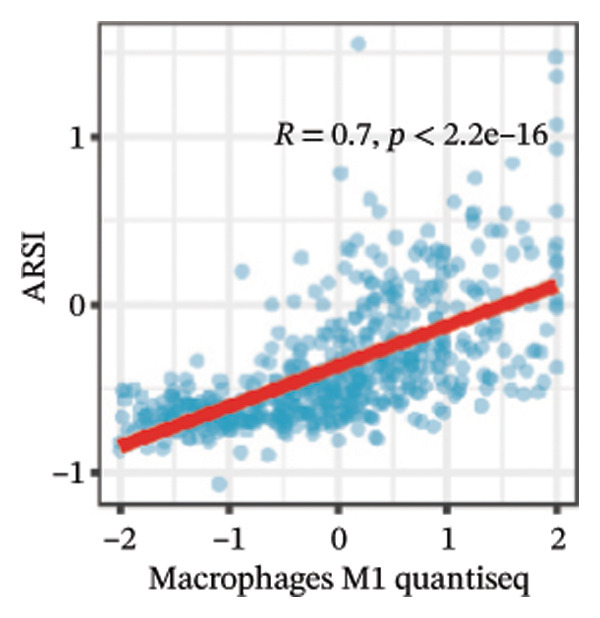
(c)

**Figure:**
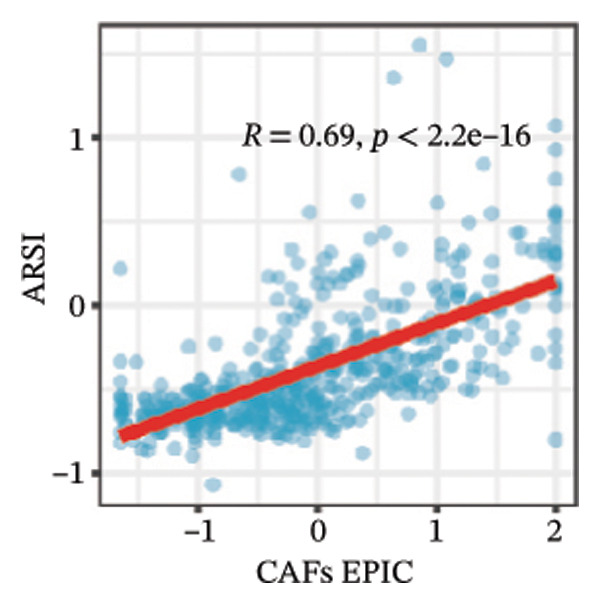
(d)

**Figure:**
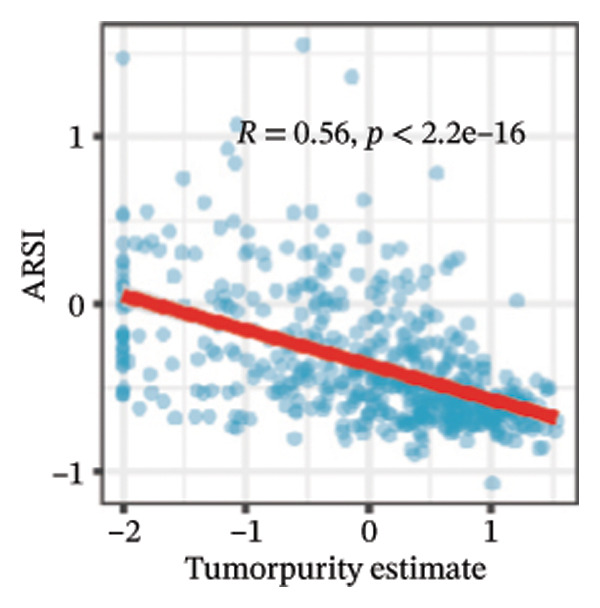
(e)

**Figure:**
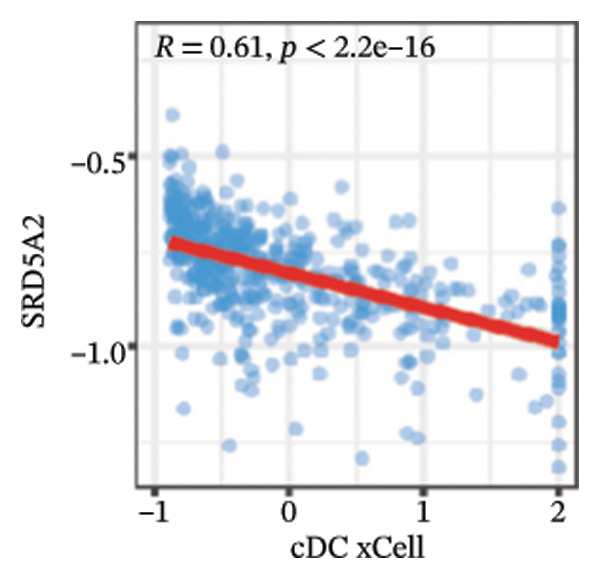
(f)

**Figure:**
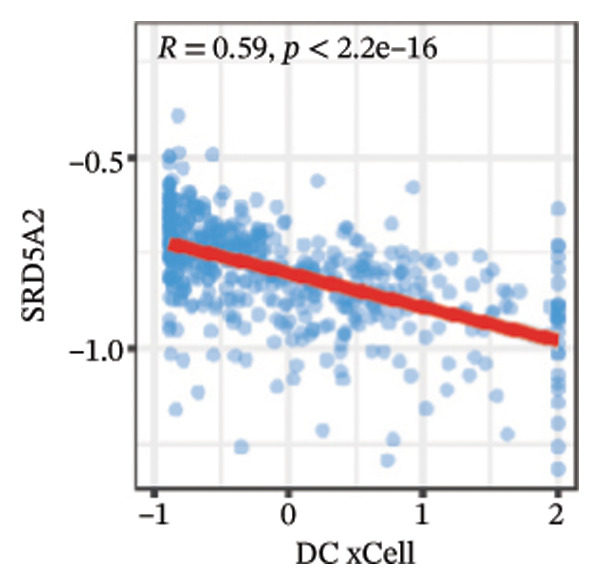
(g)

**Figure:**
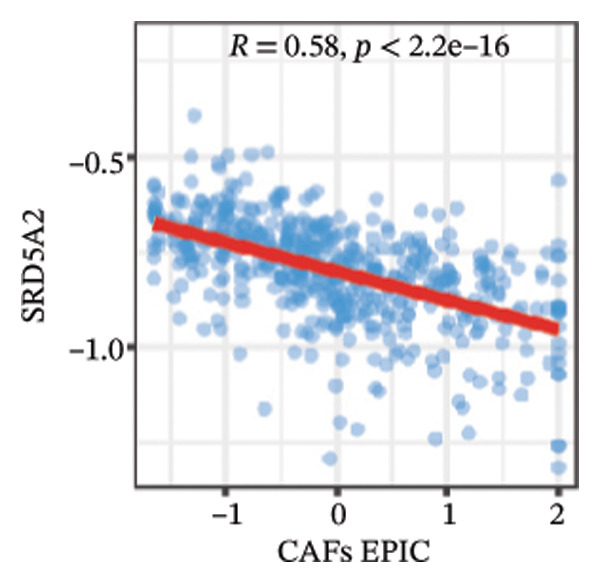
(h)

**Figure:**
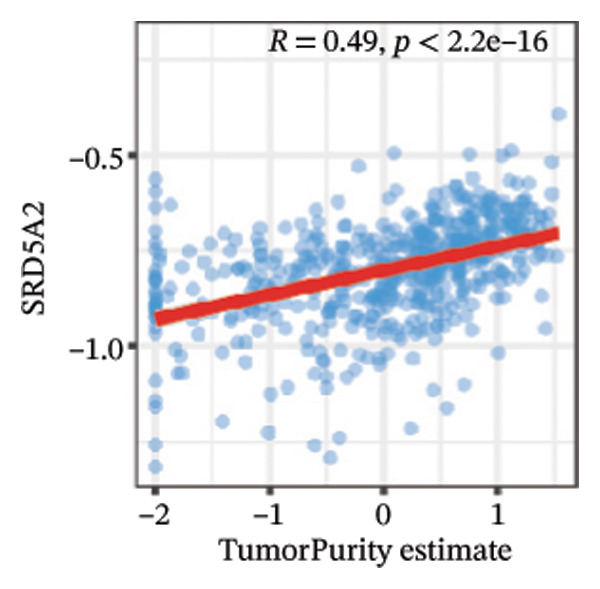
(i)

**Figure:**
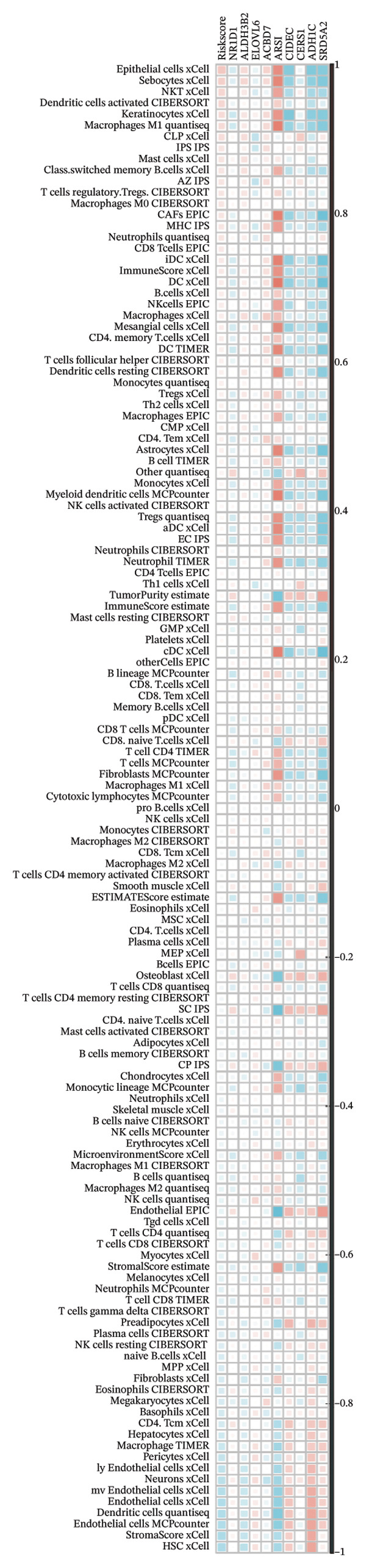
(j)

### 3.7. Drug Sensitivity of Risk Model Genes

After screening, we identified significant associations between four genes (ALDH3B2, ACBD7, CIDEC, and CERS1) and sensitivity to 11 drugs, with absolute correlation coefficients of these associations greater than 0.55 and *p* values of less than 0.01. Specific results showed that ALDH3B2 was positively correlated with sensitivity to five drugs, including Fulvestrant, SR16157, VT.464, AZD.9496, and GDC.0810 (see Figures 7(a), 7(b), 7(c), 7(d), 7(e)); ACBD7 was positively correlated with the sensitivity of three drugs, namely, methylprednisolone, dexamethasone (Decadron), and Fludarabine.1 (see Figures 7(f), 7(g), 7(h), 7(i)); and CIDEC was negatively associated with drug sensitivity to Olmutinib (see Figure 7(j)), while CERS1 was negatively associated with drug sensitivity to ON.123300 (see Figure 7(k)).

FIGURE 7Associations between risk‐model genes and predicted drug sensitivity. (a–e) Positive correlations between ALDH3B2 expression and sensitivity to Fulvestrant, SR16157, VT‐464, AZD‐9496, and GDC‐0810. (f–i) Positive correlations between ACBD7 expression and sensitivity to methylprednisolone, dexamethasone (Decadron), Fludarabine, and Nelarabine. (j) Negative correlation between CIDEC expression and sensitivity to Olmutinib. (k) Negative correlation between CERS1 expression and sensitivity to ON.123300.

**Figure:**
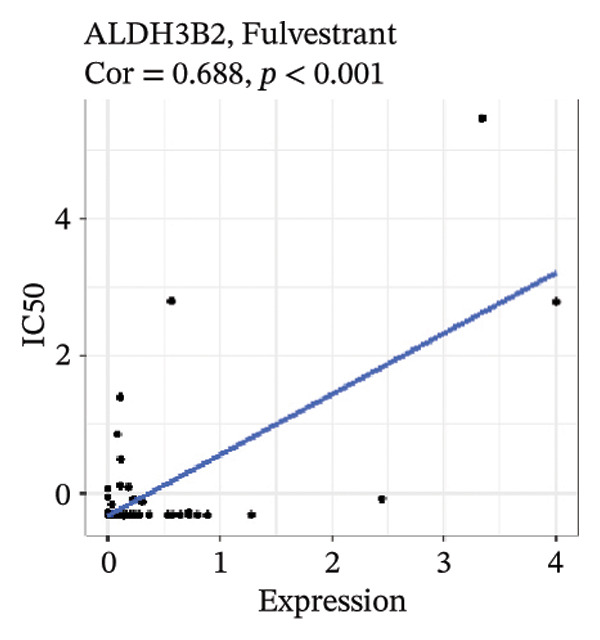
(a)

**Figure:**
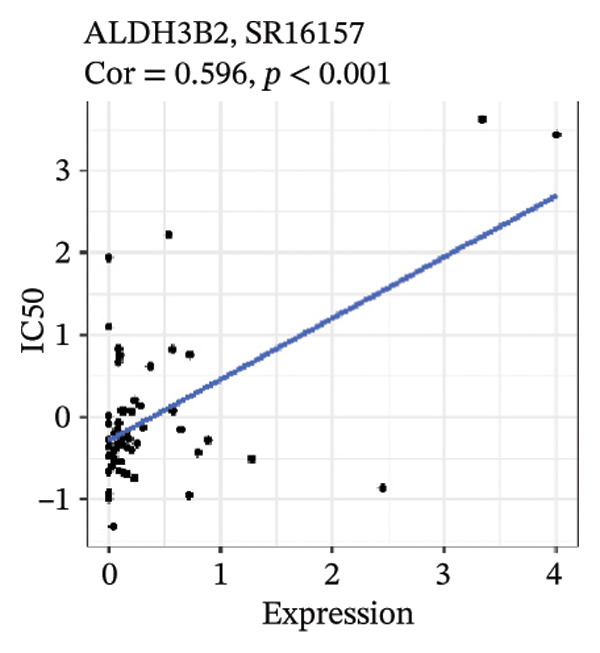
(b)

**Figure:**
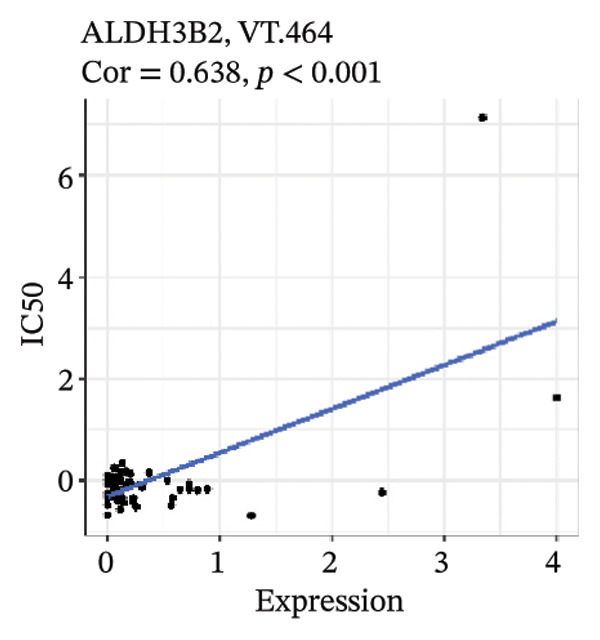
(c)

**Figure:**
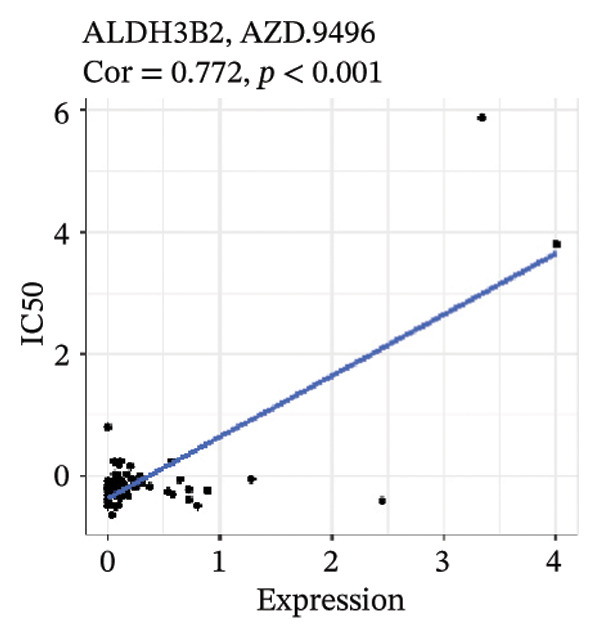
(d)

**Figure:**
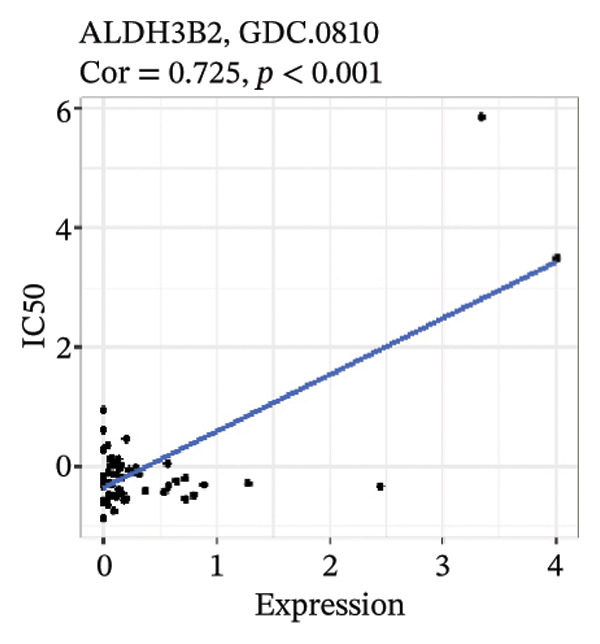
(e)

**Figure:**
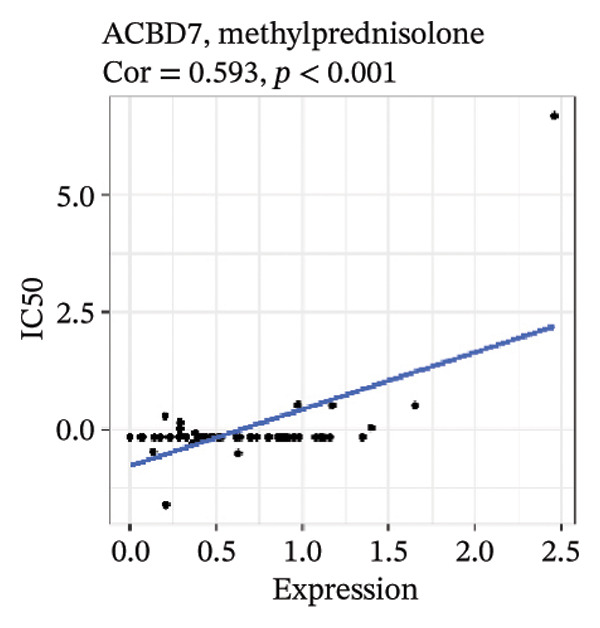
(f)

**Figure:**
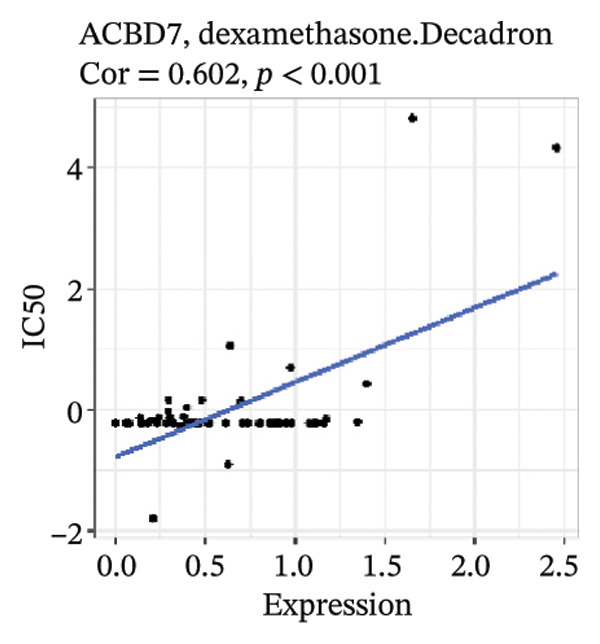
(g)

**Figure:**
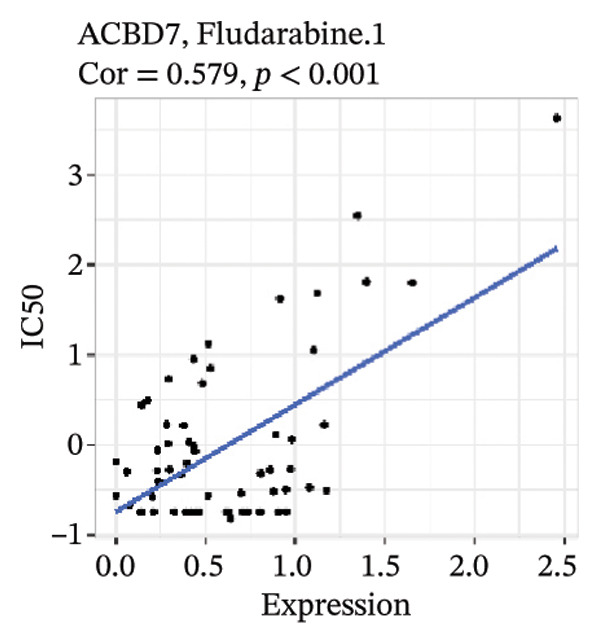
(h)

**Figure:**
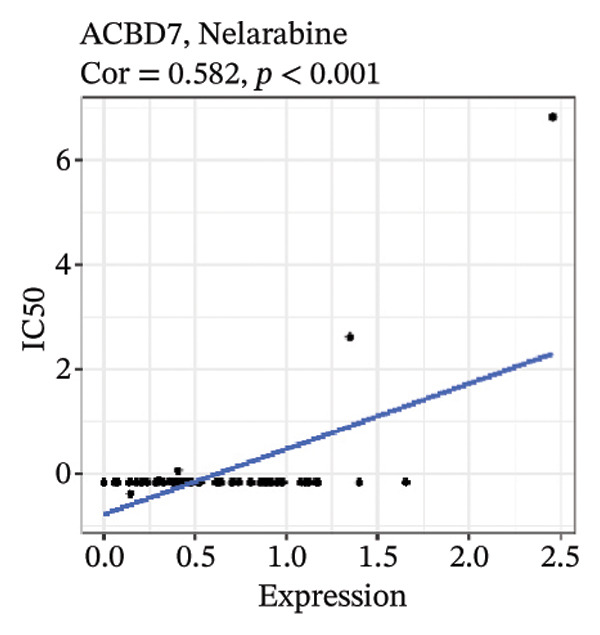
(i)

**Figure:**
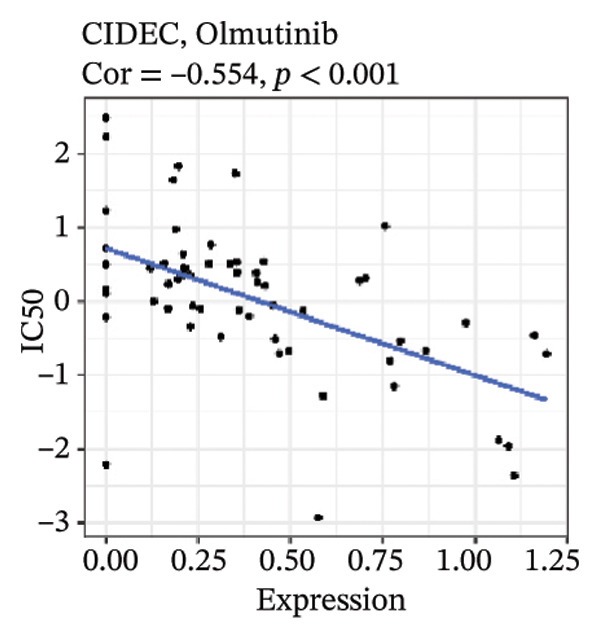
(j)

**Figure:**
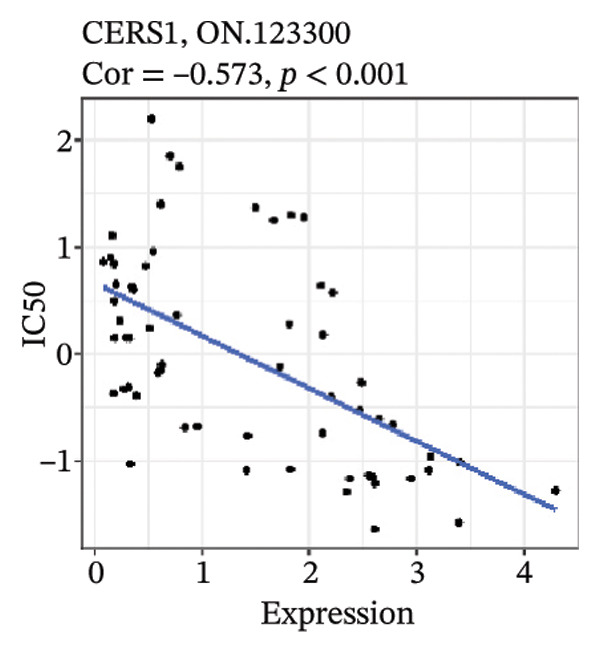
(k)

### 3.8. RiskScore Combined With Clinicopathological Features to Further Improve Prognostic Modeling and Survival Prediction

Finally, in both the training and validation subsets, multivariate Cox models that included age, sex, and tumor stage showed that RiskScore remained an independent prognostic factor (Figures 8(a) and 8(b)). To quantify the risk assessment and survival probability of patients, we combined RiskScore and other clinicopathological characteristics to create a column‐line graph as in Figure 8(c), which showed that RiskScore had the greatest impact on survival prediction from the model results. We compared the AUC values from 3 to 10 years and found that the column chart model had better prediction performance than other features after 5 years (Figure 8(d)). We further evaluate the prediction accuracy of the model by using the calibration curve as shown in Figure 8(e), and we can observe that the prediction calibration curves of the three calibration points at 3, 5, and 8 years are close to coinciding with the standard curves, which suggests that the column charts have good prediction performance. In addition, we also assessed the reliability of the model using decision curve analysis (DCA), and it can be observed that both RiskScore and nomogram benefits are significantly higher than the extreme curves, and both the nomogram and RiskScore show the strongest survival prediction performance compared to other clinicopathological features Figure 8(f).

FIGURE 8Integration of the RiskScore with clinical variables to build and evaluate a prognostic nomogram. (a) Univariate Cox regression analysis of the RiskScore and major clinical characteristics. (b) Multivariate Cox regression identifying independent prognostic factors after adjusting for clinical covariates. (c) Nomogram incorporating the RiskScore and clinical variables to predict overall survival at multiple time points. (d) Time‐dependent AUC curves demonstrating the predictive performance of the nomogram compared with individual clinical factors. (e) Calibration curves evaluating the agreement between nomogram‐predicted and observed survival at 3, 5, and 8 years. (f) Decision curve analysis (DCA) assessing the clinical utility of the nomogram relative to other predictors.

**Figure:**
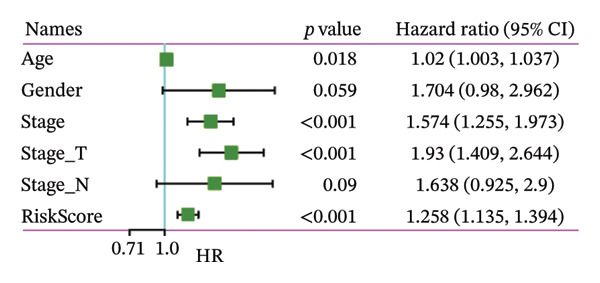
(a)

**Figure:**
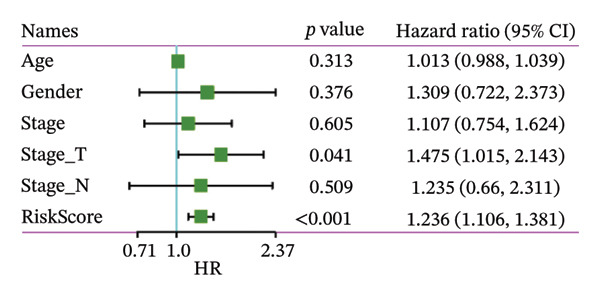
(b)

**Figure:**
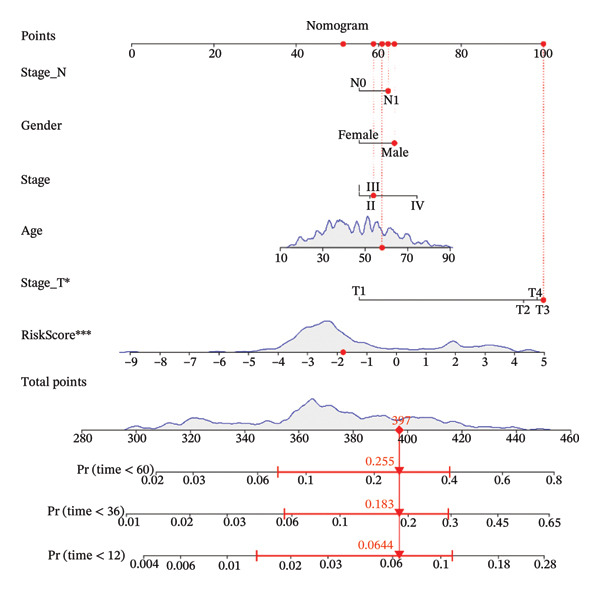
(c)

**Figure:**
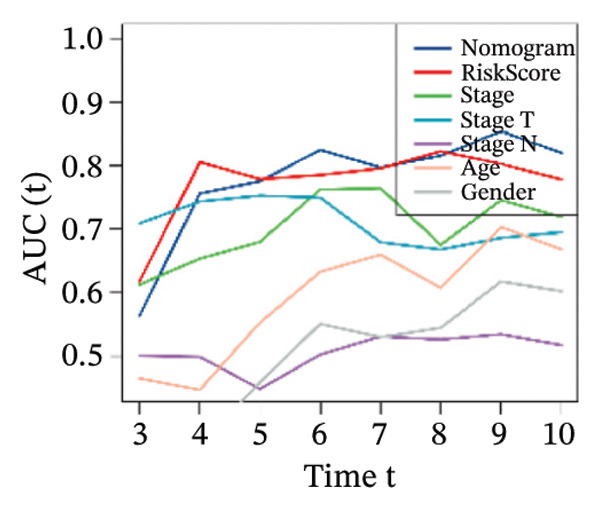
(d)

**Figure:**
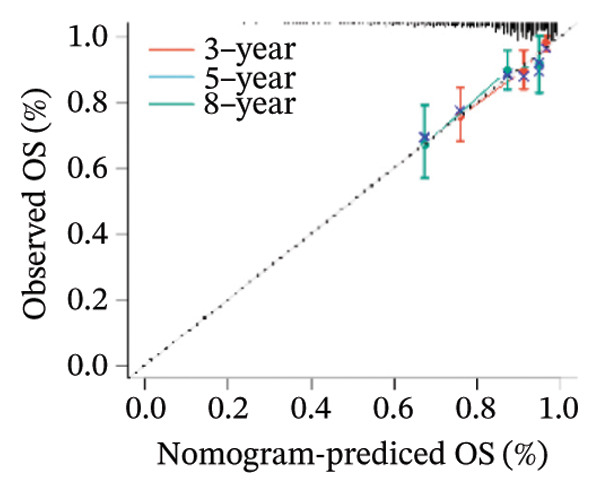
(e)

**Figure:**
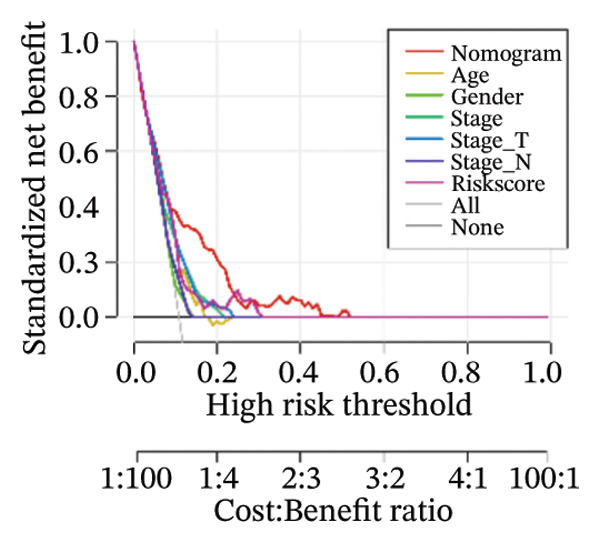
(f)

### 3.9. Transcriptional Status of Risk Genes: High Activity Genes

Through rigorous cell quality control, we finally obtained 108,741 cells and annotated them into 10 different cell types, such as T/NK cells, epithelial cells, monocyte macrophages, and so on (see Figure 9(a) and Supporting Figure 6). We also show the percentage of each cell type in each sample (see Figure 9(b)). In addition to this, for the upregulated differential genes for each cell type, we performed differential gene and enrichment analyses and visualized the results as a heat map (see Figure 9(c)).

FIGURE 9Cell‐type annotation and transcriptional activity of lipid‐metabolism–related risk genes in thyroid cancer single‐cell data. (a) UMAP visualization of the annotated cell populations from thyroid cancer single‐cell RNA‐seq samples, including mononuclear phagocytes, T/NK cells, pDCs, B/plasma cells, endothelial cells, epithelial cells, fibroblasts, pericytes, and others. (b) Proportion of each annotated cell type in individual thyroid cancer samples. (c) Heatmap displaying representative marker genes and enriched KEGG pathways across cell types, highlighting transcriptional programs associated with lipid metabolism. (d) UMAP plot indicating the distribution of cells with high versus low activity of the lipid‐related risk gene signature. (e) GSEA plots showing enrichment of glycerolipid and glycerophospholipid metabolism pathways in cells with high‐risk gene activity.

**Figure:**
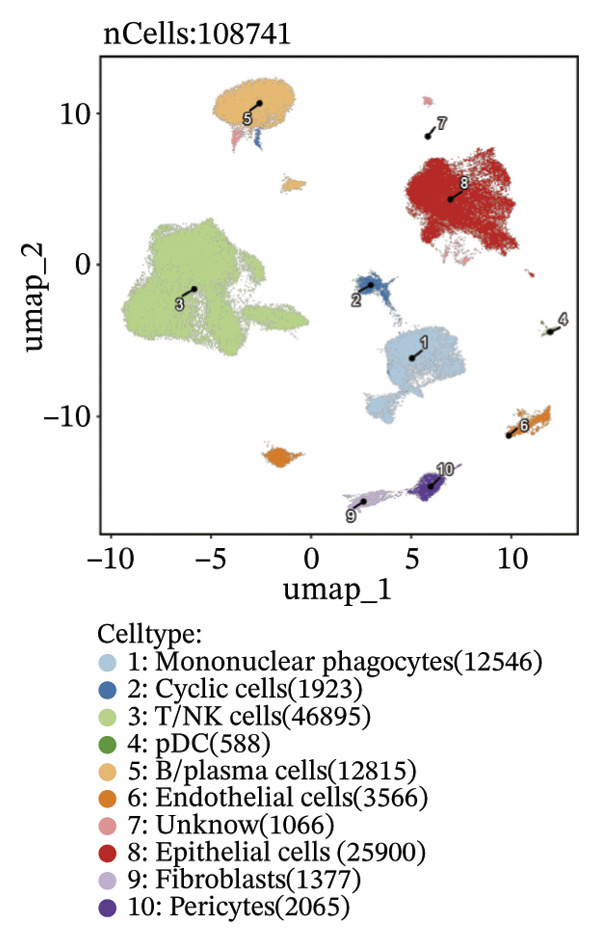
(a)

**Figure:**
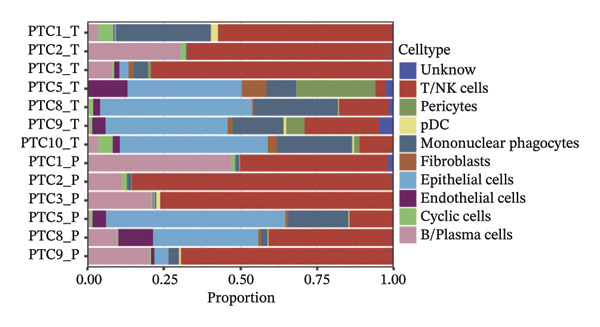
(b)

**Figure:**
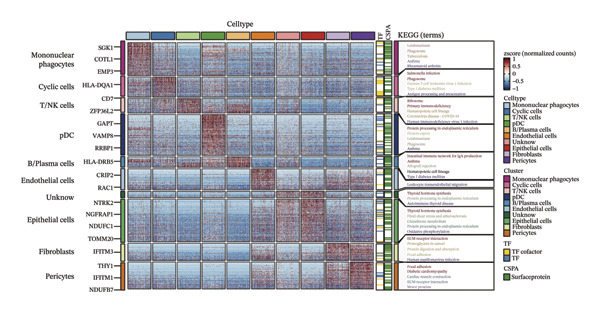
(c)

**Figure:**
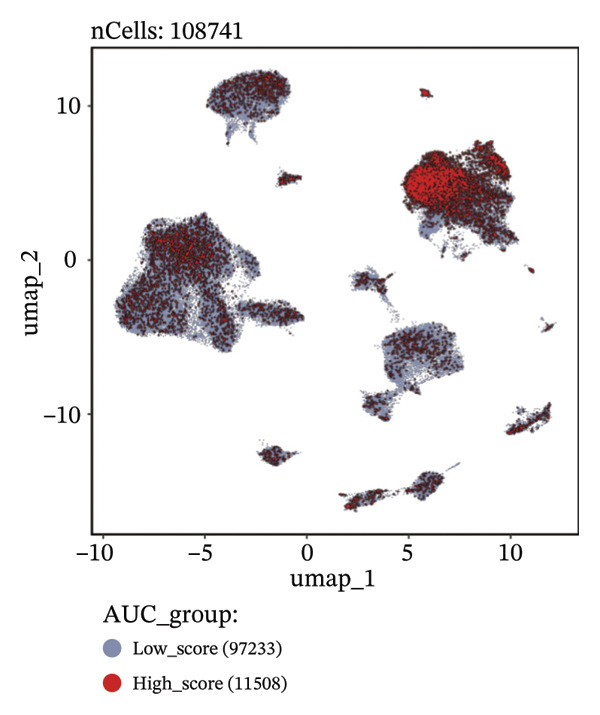
(d)

**Figure:**
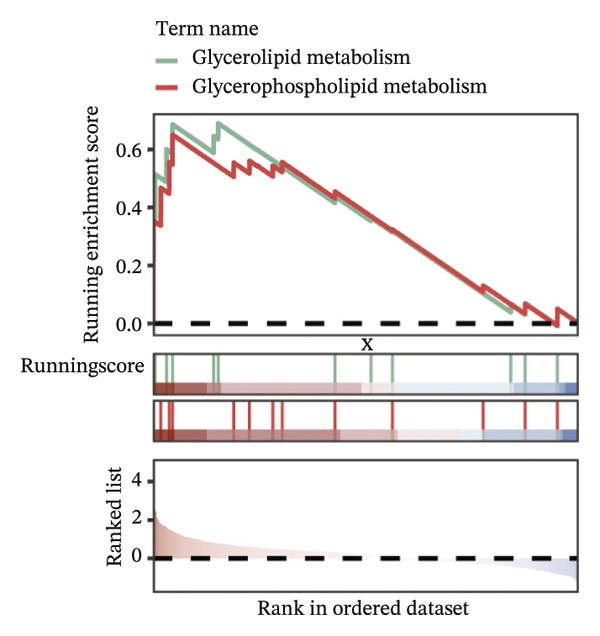
(e)

Using the AUCell algorithm to determine a scoring threshold of 0.02, we identified a total of 11,508 cells with high activity of risk genes (see Figure 9(d)). Differential gene analysis was further performed on high‐ and low‐activity cells, and GSEA enrichment analysis revealed significant activation of the glycerolipid metabolism and glycerophospholipid metabolism pathways in the high‐activity group (see Supporting Figure 7 and Figure 9(e)).

### 3.10. Function Analysis of ACBD7 in Thyroid Cancer

To investigate the impact of ACBD7 on thyroid cancer cell behavior, clone formation and transwell assays were conducted to examine its regulatory functions. The findings demonstrated that suppressing ACBD7 expression significantly reduced the proliferative capacity (Figure 10(a)) and decreased the invasion and migration abilities of TPC‐1 cells in vitro (Figure 10(b)).

FIGURE 10Functional effects of ACBD7 knockdown in thyroid cancer cells. (a) Colony formation assay showing reduced proliferative capacity of TPC‐1 cells following ACBD7 knockdown (si‐ACBD7) compared with negative control (si‐NC). ^∗∗∗^ *p* < 0.001. (b) Transwell migration assay demonstrating that ACBD7 knockdown markedly decreases the migratory and invasive ability of TPC‐1 cells. ^∗∗∗^ *p* < 0.001.

**Figure:**
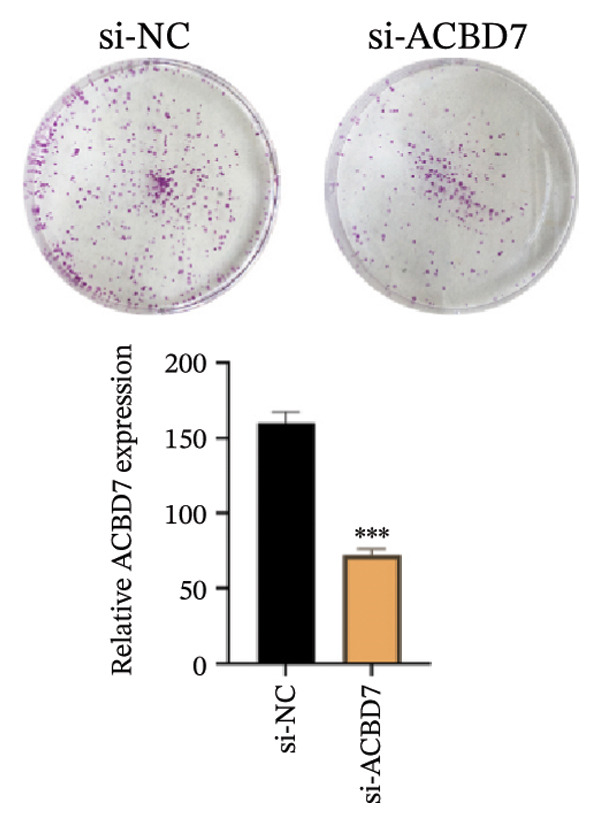
(a)

**Figure:**
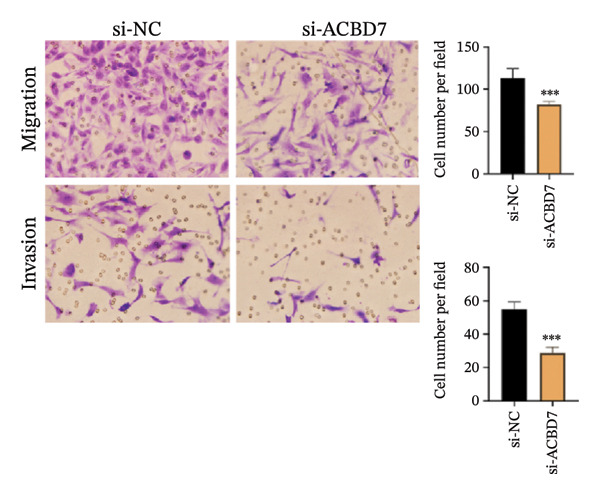
(b)

## 4. Discussion

The epidemiology of thyroid cancer has shown a significant increase in incidence rates globally, with PTC being the most common type, while ATC remains one of the most aggressive forms [33]. Despite current treatment options such as surgery, radioiodine therapy, and chemotherapy, there is a lack of effective therapeutic targets for advanced or refractory thyroid cancer, particularly ATC.

Our study adds to the growing body of research on potential therapeutic targets by focusing on the role of lipid metabolism in thyroid cancer. From screening over 400 metabolites, we identified lipids as the largest class associated with thyroid cancer progression. Through differential analysis, 80 lipid metabolism–related genes were found to be upregulated, while 40 were downregulated. We incorporated nine key lipid metabolism–related genes, including ACBD7, into a prognostic risk model that effectively predicted patient outcomes. The genomic differences between high‐ and low‐risk groups revealed significant alterations in lipid metabolic pathways, such as glycerolipid and glycerophospholipid metabolism, which were more active in the high‐risk group. Immune landscape analysis showed that certain immune cell populations, like macrophages and DC, were significantly associated with risk model genes. The differences in immune infiltration patterns between risk groups may also have biological and clinical implications. The high‐risk group showed increased DC, M1‐like macrophages, and CAF enrichment, which are features often linked to stromal remodeling and impaired antitumor immunity. These changes may create a microenvironment that supports tumor growth and weakens T‐cell function, suggesting a possible mechanism of immune evasion.

Beyond these transcriptomic and immune differences, the causal metabolites identified through MR offer a mechanistic explanation for why lipid‐related pathways are altered in thyroid tumors. Metabolites with strong genetic support can activate downstream lipid‐handling enzymes and transcriptional regulators through substrate‐driven metabolic signaling. Such signals influence membrane remodeling, fatty‐acid turnover, and arachidonic‐acid–related pathways, all of which were enriched in the high‐risk group. These mechanisms provide a genetic basis for the transcriptional shifts we observed and suggest that inherited metabolic predisposition may shape the lipid metabolic program of thyroid tumors.

ACBD7‐associated lipid changes may further contribute to immune escape. Increased lipid synthesis can generate fatty‐acid–derived mediators that influence macrophage behavior. Such signals may push tumor‐associated macrophages toward pro‐tumor polarized states, reduce antigen handling, and weaken local T‐cell activity. These effects are consistent with the higher macrophage infiltration we observed in high‐risk tumors.

ARSI also showed strong correlations with CAFs and other stromal populations. High ARSI expression marked samples with greater stromal content, suggesting a role in extracellular‐matrix remodeling. Stromal expansion can limit immune‐cell access, reinforce immunosuppressive niches, and support tumor persistence.

Taken together, ACBD7‐driven lipid remodeling and ARSI‐associated stromal activation may act in parallel to create a microenvironment that favors immune evasion. This provides a mechanistic explanation for the coordinated metabolic and immunologic features seen in high‐risk tumors.

Lipid metabolic activity may also shape the immune response in endocrine tumors. Thyroid cancer cells with enhanced lipid synthesis can produce bioactive lipids that influence cytokine release and dendritic cell behavior. These lipids may push macrophages toward inflammatory but tumor‐supportive states or reduce antigen presentation. Such effects weaken local T‐cell activity and create an immune environment that favors tumor persistence.

Endocrine tumors often rely on flexible metabolic programs. When lipid pathways are upregulated, the resulting metabolites can act as immune‐modulating signals, linking metabolic reprogramming with immune escape. This mechanism offers a possible explanation for why high‐risk tumors with stronger lipid pathway activation also show distinct immune infiltration patterns.

The mutation landscape further supports this interpretation. The higher BRAF mutation rate and increased nonsilent mutation burden in the high‐risk group are hallmarks of more aggressive tumor behavior and may indicate a higher degree of genomic instability. Such features can influence immunotherapy response, as tumors with strong stromal activation but moderate mutation burden sometimes show limited benefit from immune checkpoint inhibitors. Together, these findings suggest that lipid‐driven metabolic alterations may contribute to a more immunosuppressive and clinically aggressive tumor phenotype.

Importantly, functional assays demonstrated that ACBD7 suppression reduced the proliferation, invasion, and migration of TPC‐1 cells, which supports the notion that lipid metabolism is integral to thyroid cancer progression. This study provides a comprehensive view of how lipid metabolism–related genes may serve as therapeutic targets in thyroid cancer, building on earlier works in related fields. Several of the pathways highlighted in our analysis, including fatty‐acid elongation, glycerophospholipid turnover, and arachidonic‐acid signaling, have emerging therapeutic relevance. Small‐molecule inhibitors targeting fatty‐acid synthase, stearoyl‐CoA desaturase, and arachidonic‐acid–producing enzymes are already being tested in solid tumors. Our findings suggest that similar metabolic vulnerabilities may exist in thyroid cancer, particularly in patients classified as high‐risk by our signature.

The nine‐gene panel identified in this study may complement current molecular markers used in thyroid cancer, such as BRAF V600E, TERT promoter mutations, and RET/NTRK fusions. Unlike these genomic drivers, lipid‐metabolism genes capture metabolic reprogramming that is not reflected by mutation status alone. This distinction may offer added value for risk stratification, especially in tumors without actionable mutations.

ACBD7 is of particular interest because suppressing its expression reduced tumor cell proliferation and invasion in our experiments. This supports the idea that lipid‐handling proteins may be druggable nodes in thyroid cancer biology. Incorporating metabolic markers such as ACBD7 into existing therapeutic frameworks could help refine patient selection for targeted therapies or guide the development of metabolism‐directed treatments.

The competing‐risk model showed stable predictive performance across multiple evaluations. Its C‐index and time‐dependent AUC values remained high, indicating reliable discrimination over time. Calibration curves showed close agreement between predicted and observed outcomes, suggesting that the model provides accurate risk estimates in both short‐term and long‐term follow‐up. DCA further demonstrated meaningful clinical net benefit compared with standard clinical factors alone. These findings support the robustness of the model and suggest that it could add value to current prognostic tools in thyroid cancer.

Previous studies have investigated the roles of ALDH3B2, ACBD7, CIDEC, and CERS1 in various cancers, revealing diverse mechanisms. For instance, ALDH3B2 has been shown to promote tumor progression through lipid oxidation pathways in hepatocellular carcinoma [34], while ACBD7 has been linked to fatty acid transport and energy regulation in colorectal cancer [35]. CIDEC, known for its role in lipid droplet formation, has been implicated in breast cancer progression, and CERS1, a key enzyme in sphingolipid metabolism, has been found to inhibit tumor growth in glioma [36]. In contrast, this study is the first to highlight these genes in the context of thyroid cancer, revealing novel oncogenic roles and therapeutic potentials that have not been reported in this malignancy. Compared to earlier findings, such as those in “Single‐cell and WGCNA uncover a prognostic model and potential oncogenes in colorectal cancer [37],” our study expands the understanding of these genes by identifying their specific involvement in thyroid cancer and their impact on lipid metabolism.

While our study provides significant insights into the role of lipid metabolism in thyroid cancer, there are limitations that need to be addressed. First, although our prognostic model was validated using both training and validation sets, larger datasets and longer follow‐up periods are necessary to further substantiate its clinical utility. Additionally, in vitro functional assays, while informative, need to be complemented with in vivo studies to confirm the therapeutic potential of targeting ACBD7 and other identified genes. Further research should also explore the interaction between lipid metabolism–related genes and the immune microenvironment, as this may unveil additional therapeutic strategies. Despite these limitations, our study offers a robust framework for understanding the metabolic underpinnings of thyroid cancer and highlights the potential for lipid metabolism–related genes to serve as novel therapeutic targets.

In conclusion, this study provides novel insights into the role of lipid metabolism–related genes in thyroid cancer progression. By integrating single‐cell sequencing, GWAS data, and in vitro functional assays, we identified key genes, such as ACBD7, that may serve as promising therapeutic targets. These findings have potential clinical implications for developing more targeted therapies for aggressive thyroid cancers, including ATC. The ability to target lipid metabolism in cancer represents a promising avenue for future therapeutic interventions. Our work contributes significantly to the growing body of research on thyroid cancer, offering new directions for personalized medicine and improving patient outcomes.

This study has several limitations. First, most genetic data used in the MR analyses were derived from European populations, which may reduce generalizability to other ancestries. Second, although transcriptomic and single‐cell datasets provided consistent results, the overall sample size remains moderate and limits subgroup analyses. Third, the functional validation was performed in vitro, and in vivo experiments are still needed to confirm the biological role of ACBD7 and to evaluate whether lipid‐metabolism pathways influence tumor behavior in a physiological microenvironment. These limitations should be addressed in future work to strengthen the clinical relevance of our findings.

Future studies should examine how ACBD7 and other lipid‐related genes regulate metabolic‐immune interactions in thyroid cancer. These experiments should track lipid flux and immune‐cell behavior in real time, ideally using co‐culture systems or spatial techniques to map how tumor cells, CAFs, and macrophages respond to lipid remodeling.

Another direction is to test whether blocking ACBD7‐associated pathways can enhance the effects of immunotherapy or targeted therapy. Preclinical models could evaluate combinations of lipid‐metabolic inhibitors with BRAF‐ or immune‐checkpoint–directed agents.

Larger prospective cohorts are also needed to validate the nine‐gene signature and determine whether it can support clinical decision‐making. Integrating lipidomics with single‐cell and imaging‐based analyses may refine this signature and reveal additional metabolic markers with prognostic or therapeutic value [38].

## Author Contributions

Conceptualization, W.Z.; methodology, W.Z.; software, T.W., B.N., and Q.S.; validation, T.W., B.N., and Q.S.; formal analysis, T.W., B.N., and Q.S.; investigation, T.W., B.N., and Q.S.; resources, T.W., B.N., and Q.S.; writing–original draft preparation, T.W., B.N., and Q.S.; writing–review and editing, W.Z.; visualization, T.W., B.N., and Q.S.; supervision, N.X. and W.Z.; project administration, N.X. and W.Z.; funding acquisition, N.X. and W.Z.

## Funding

This work was supported by the Natural Science Foundation of Liaoning Province (Grant No. 24‐214‐3‐25).

## Disclosure

All authors have read and agreed to the published version of the manuscript.

## Ethics Statement

The authors have nothing to report.

## Consent

The authors have nothing to report.

## Conflicts of Interest

The authors declare no conflicts of interest.

## Supporting Information

Additional supporting information can be found online in the Supporting Information section.

## Supporting information


**Supporting Information 1** Supporting Figure 1. This figure shows the IVW estimates of significant blood metabolites associated with thyroid cancer. Black squares represent the IVW estimates, and black bars indicate the 95% confidence intervals of the IVW estimates. A ratio greater than 1 indicates an increased risk, while a ratio less than 1 suggests a reduced risk.

**Supporting Information 2** Supporting Figure 2. The scatter plot demonstrates the causal relationship between each metabolite and thyroid cancer, with trends validated in at least five different algorithms.

**Supporting Information 3** Supporting Figure 3. The funnel plot illustrates the effect trends across various Mendelian randomization methods, with no significant outliers observed.

**Supporting Information 4** Supporting Figure 4. This figure evaluates whether any specific SNP significantly alters the results by systematically removing individual SNPs.

**Supporting Information 5** Supporting Figure 5. Forest plot of the 14 genes identified through univariate Cox regression analysis.

**Supporting Information 6** Supporting Figure 6. Quality control and dimensionality reduction of single‐cell data. (A): Scatter plots showing the correlation between gene features (nFeature_RNA), total RNA count (nCount_RNA), and mitochondrial gene percentage (percent.mt). (B): Violin plots showing the distribution of key metrics (nFeature_RNA, nCount_RNA, percent.mt, etc.) across different samples. (C): Additional violin plots assessing sample quality using various metrics. (D): PCA and Harmony plots showing dimensionality reduction and batch effect correction, with a visualization of the data distribution and standard deviation.

**Supporting Information 7** Supporting Figure 7. Determination of thresholds for high and low activity cells based on AUCell scoring.

**Supporting Information 8** Supporting Information. STROBE Checklist.

## Data Availability

The data supporting the findings of this study are publicly available. GWAS data for blood metabolites can be accessed from the Metabolomics GWAS server (https://metabolomics.helmholtz-muenchen.de/gwas/), and thyroid tumor GWAS data are available in the GWAS catalog (https://www.ebi.ac.uk/gwas/home). The TCGA–THCA gene expression data can be accessed from The TCGA portal (https://portal.gdc.cancer.gov/). The single‐cell data used in this study are available in the GEO database (GSE184362). Additional data related to the study are available from the corresponding author upon reasonable request.
