# Supplementary figures and images for: Lipid Metabolism–Related Genes Define Prognosis and Therapeutic Targets in Thyroid Cancer
Source: Int J Genomics. 2026 May 17;2026:9661210. doi: 10.1155/ijog/9661210 (PMC13181148; doi:10.1155/ijog/9661210)

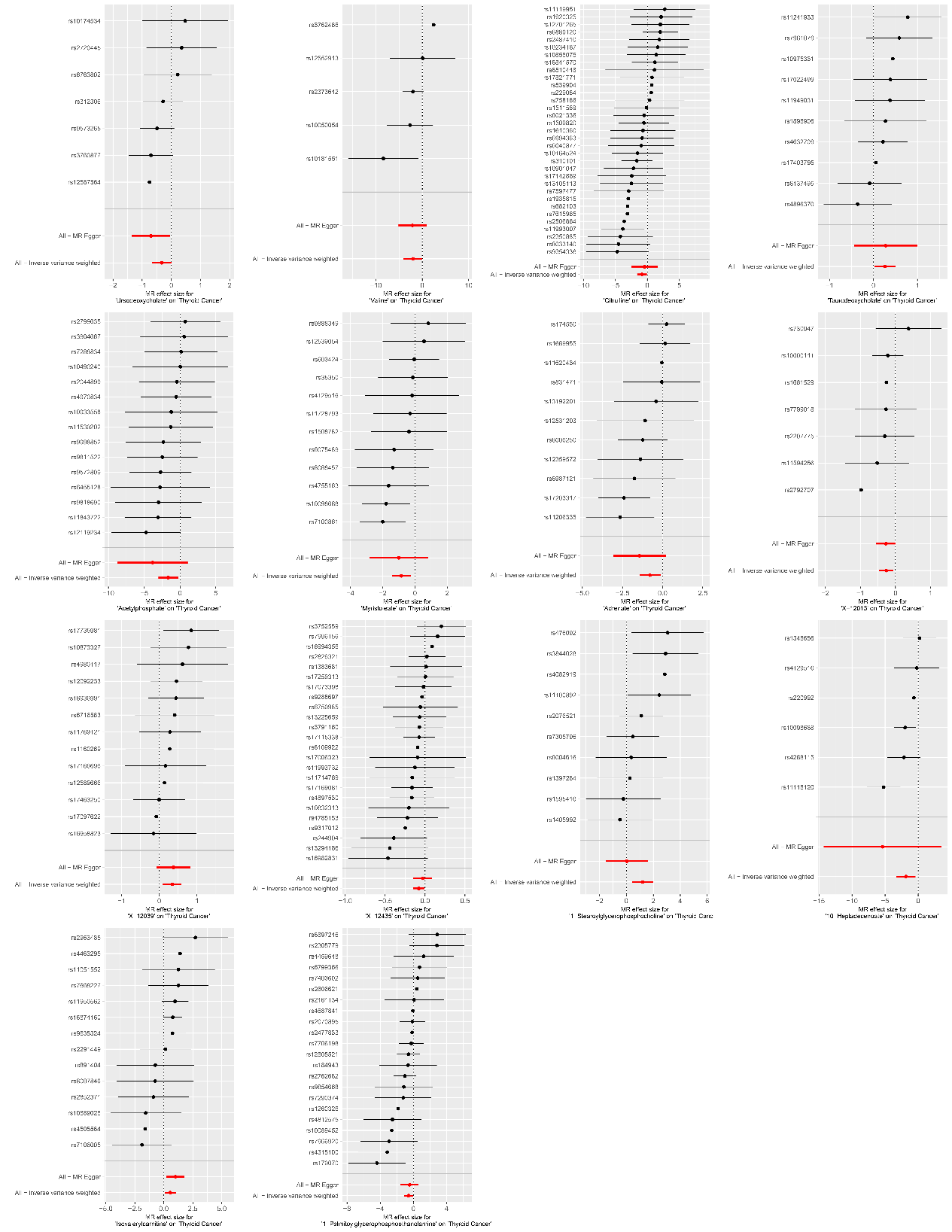

Supplement: Supplementary file 1 — Supporting Information 1 Supporting Figure 1. This figure shows the IVW estimates of significant blood metabolites associated with thyroid cancer. Black squares represent the IVW estimates, and black bars indicate the 95% confidence intervals of the IVW estimates. A ratio greater than 1 indicates an increased risk, while a ratio less than 1 suggests a reduced risk. [file IJOG-2026-9661210-s001.tif]

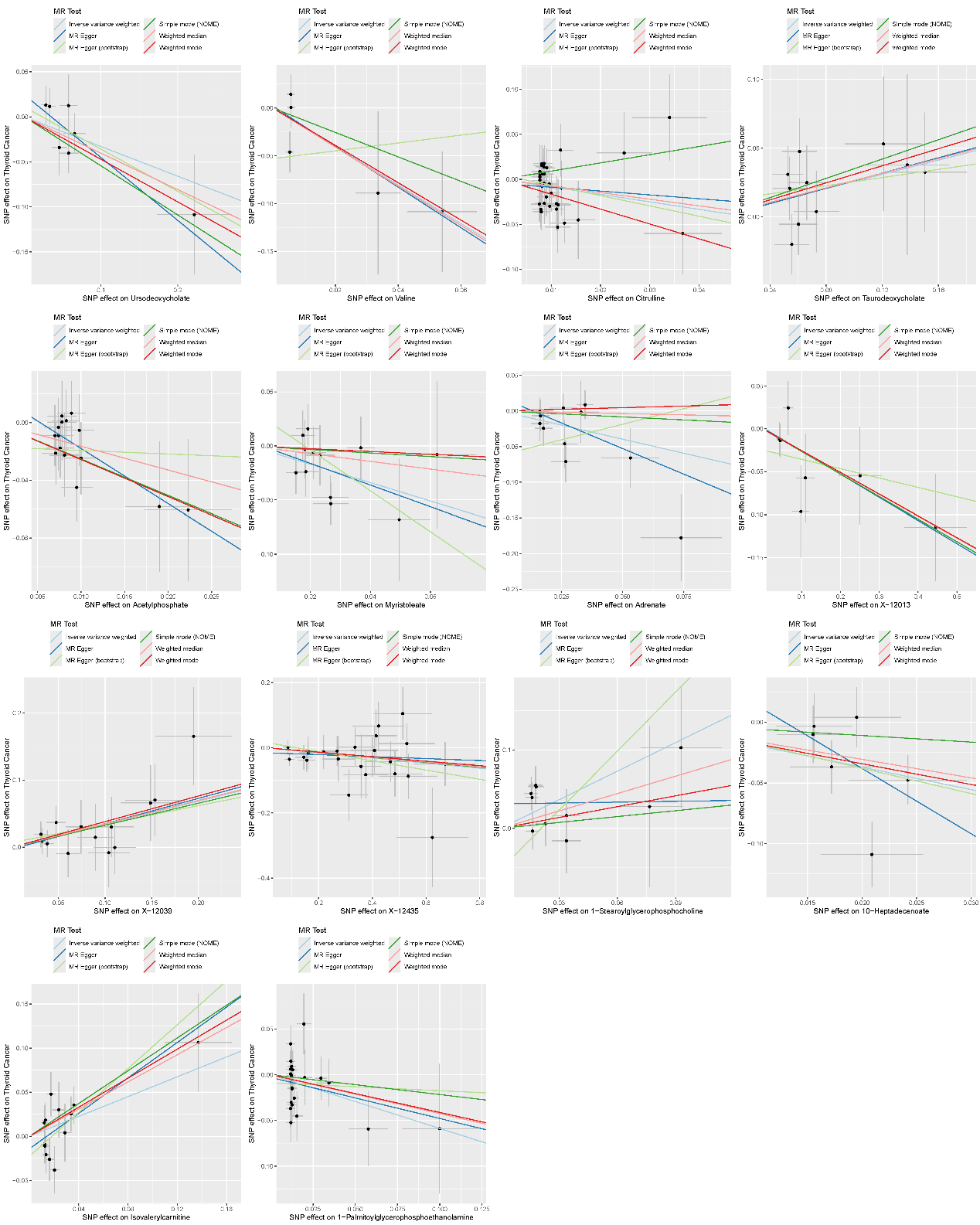

Supplement: Supplementary file 2 — Supporting Information 2 Supporting Figure 2. The scatter plot demonstrates the causal relationship between each metabolite and thyroid cancer, with trends validated in at least five different algorithms. [file IJOG-2026-9661210-s002.tif]

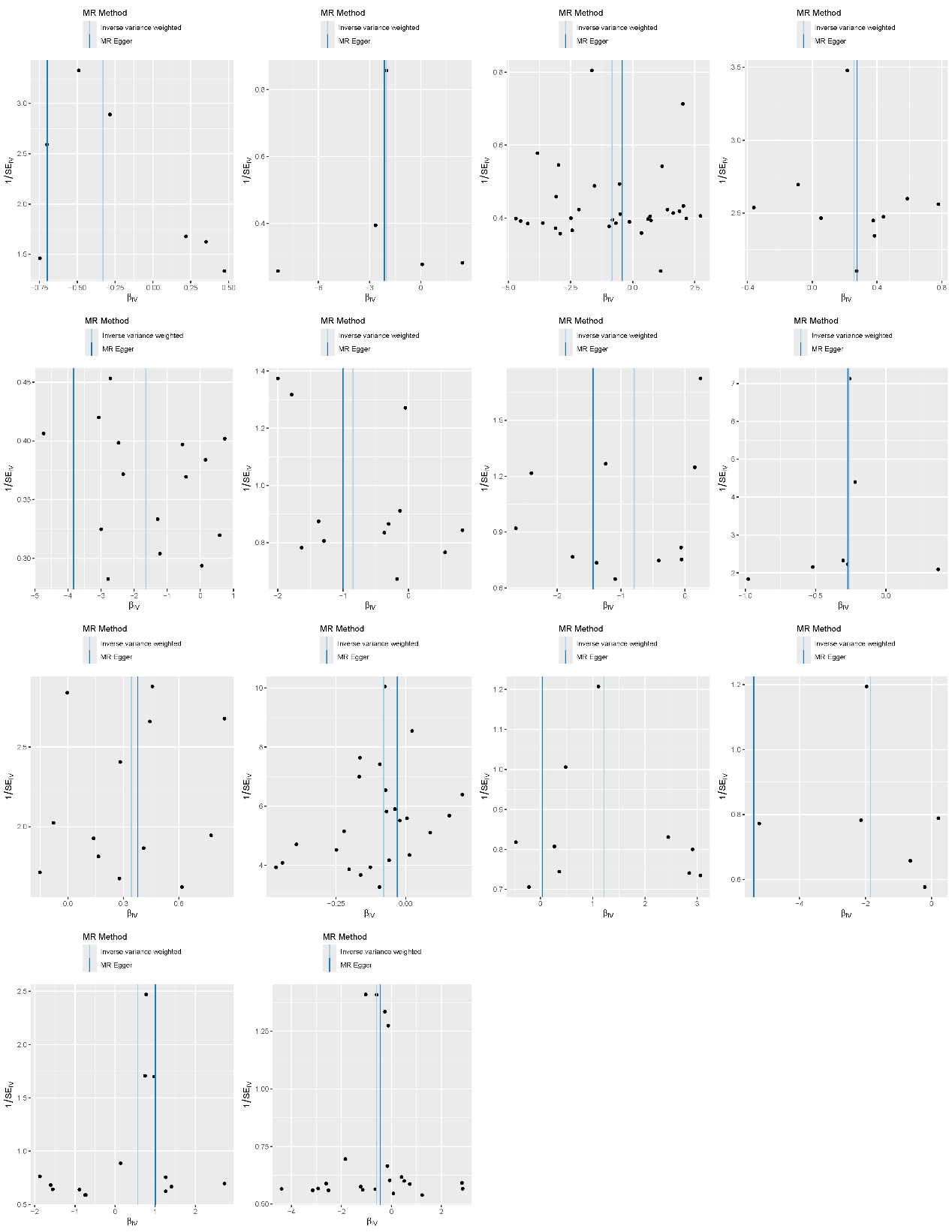

Supplement: Supplementary file 3 — Supporting Information 3 Supporting Figure 3. The funnel plot illustrates the effect trends across various Mendelian randomization methods, with no significant outliers observed. [file IJOG-2026-9661210-s003.tif]

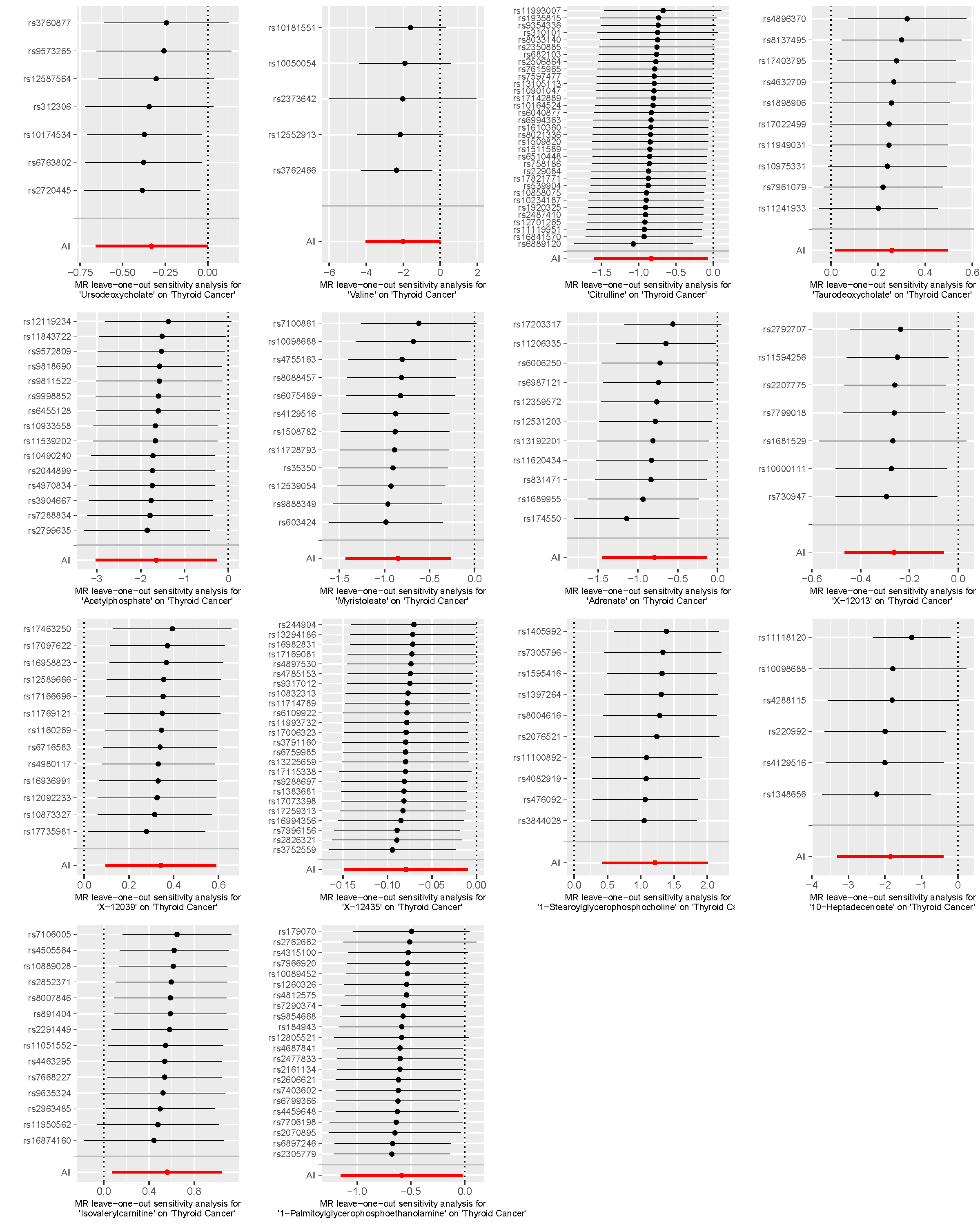

Supplement: Supplementary file 4 — Supporting Information 4 Supporting Figure 4. This figure evaluates whether any specific SNP significantly alters the results by systematically removing individual SNPs. [file IJOG-2026-9661210-s008.tif]

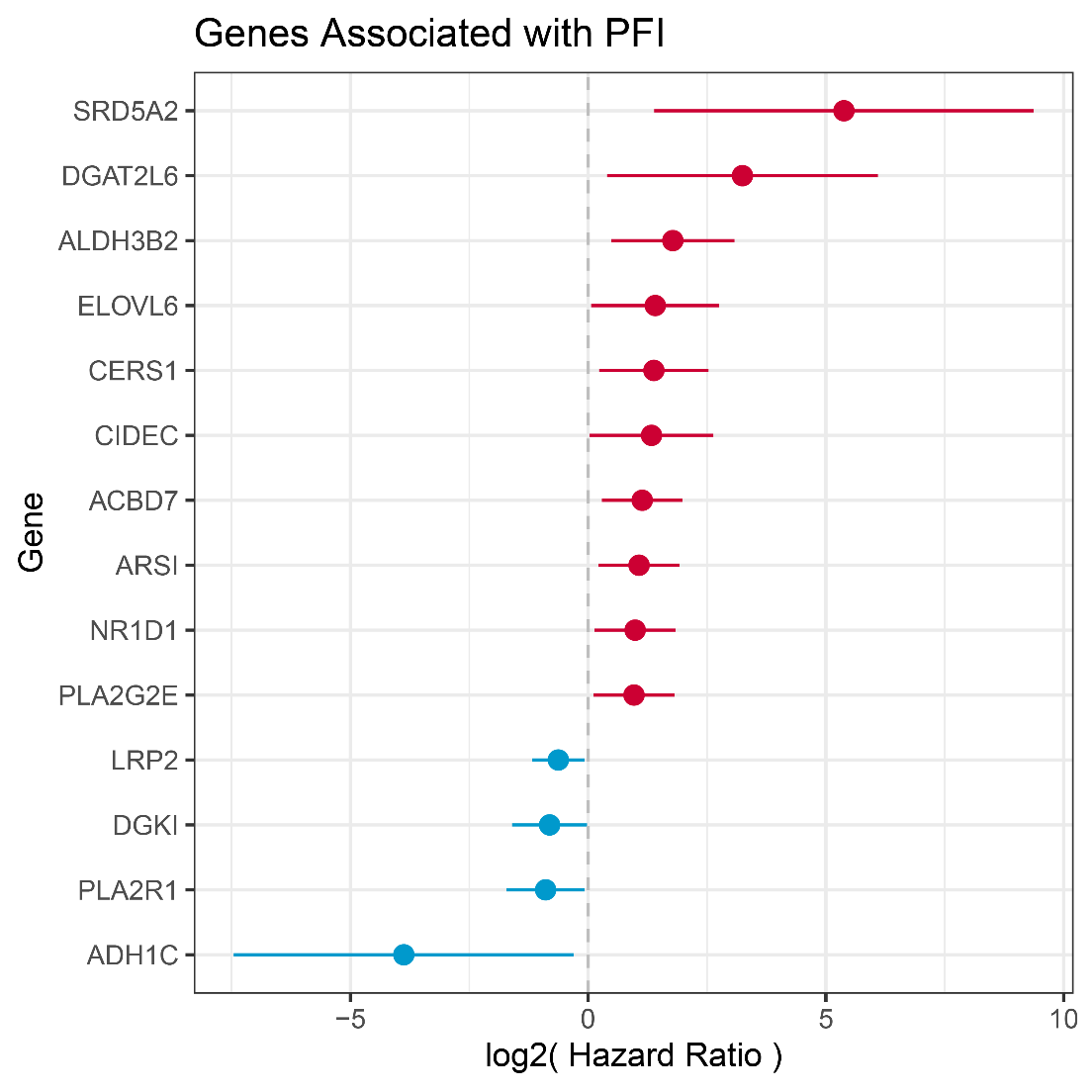

Supplement: Supplementary file 5 — Supporting Information 5 Supporting Figure 5. Forest plot of the 14 genes identified through univariate Cox regression analysis. [file IJOG-2026-9661210-s005.tif]

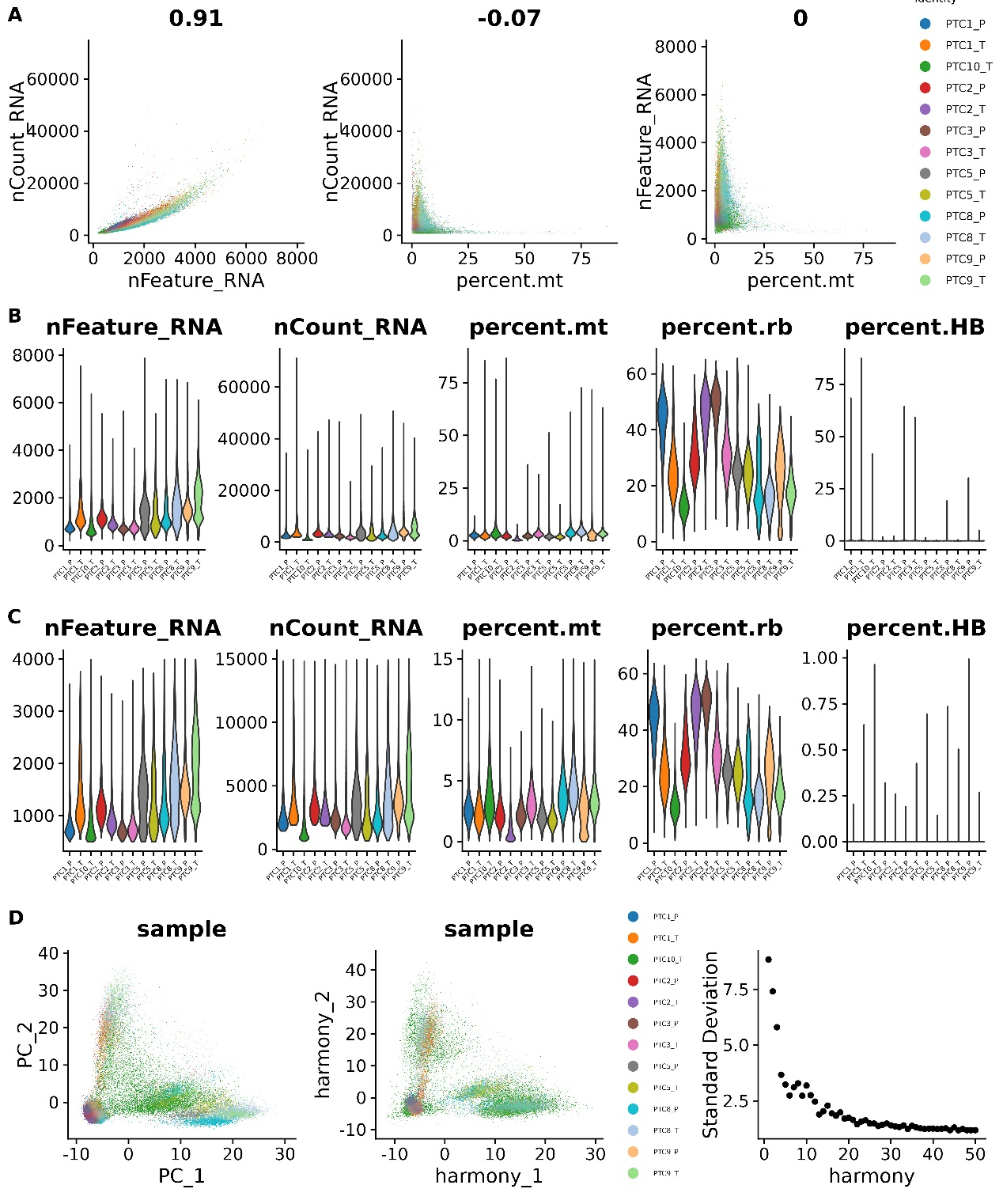

Supplement: Supplementary file 6 — Supporting Information 6 Supporting Figure 6. Quality control and dimensionality reduction of single‐cell data. (A): Scatter plots showing the correlation between gene features (nFeature_RNA), total RNA count (nCount_RNA), and mitochondrial gene percentage (percent.mt). (B): Violin plots showing the distribution of key metrics (nFeature_RNA, nCount_RNA, percent.mt, etc.) across different samples. (C): Additional violin plots assessing sample quality using various metrics. (D): PCA and Harmony plots showing dimensionality reduction and batch effect correction, with a visualization of the data distribution and standard deviation. [file IJOG-2026-9661210-s006.tif]

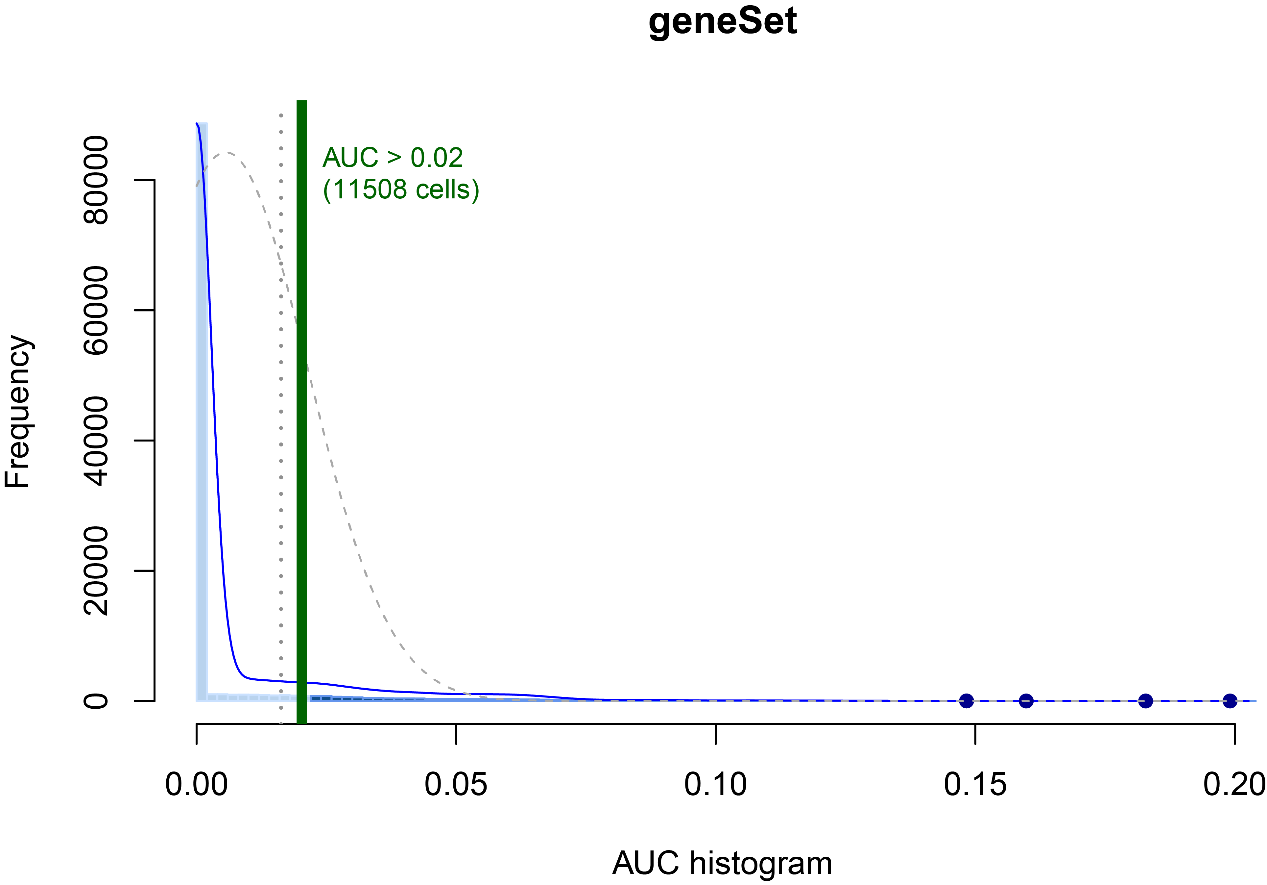

Supplement: Supplementary file 7 — Supporting Information 7 Supporting Figure 7. Determination of thresholds for high and low activity cells based on AUCell scoring. [file IJOG-2026-9661210-s007.tif]
